# Chronic Myeloid Leukemia Beyond BCR::ABL1 Inhibition: Resistance Mechanisms and Emerging Therapeutic Modalities

**DOI:** 10.3390/ph19101511

**Published:** 2026-09-23

**Authors:** Rositsa Mihaylova

**Affiliations:** Department of Pharmacology, Pharmacotherapy and Toxicology, Faculty of Pharmacy, Medical University of Sofia, 2 Dunav Str., 1000 Sofia, Bulgaria; rmihaylova@pharmfac.mu-sofia.bg

**Keywords:** chronic myeloid leukemia, BCR::ABL1, tyrosine kinase inhibitors, drug resistance, kinase mutations, leukemic stem cells, PROTACs, siRNA, adoptive cell therapy, CAR-T

## Abstract

The BCR::ABL1 fusion oncoprotein is the molecular hallmark and principal oncogenic driver of chronic myeloid leukemia (CML). Tyrosine kinase inhibitors (TKIs) have transformed CML into a largely manageable chronic disease, yet acquired resistance, persistence of leukemic stem cells (LSCs), and disease progression remain important therapeutic challenges. This narrative review examines the molecular and cellular determinants of TKI resistance and their implications for current and emerging therapeutic strategies. Resistance involves both BCR::ABL1-dependent and -independent mechanisms, ranging from kinase-domain mutations to adaptive signaling, altered drug transport, microenvironmental protection, metabolic reprogramming, and epigenetic dysregulation. Increasing understanding of these processes has yielded therapeutic strategies with growing clinical relevance, including next-generation TKIs, dual-site BCR::ABL1 inhibition, as well as rational combination approaches targeting alternative signaling and other survival dependencies. At the experimental frontier, PROTAC-mediated protein degradation, siRNA-based gene silencing, and CAR-based cellular immunotherapies are being explored as innovative approaches for targeting resistant leukemic populations. Nanotechnology-based delivery systems further provide opportunities for targeted delivery of TKIs and nucleic-acid therapeutics. In parallel, molecular monitoring and multi-omic profiling offer opportunities to better define disease evolution and guide individualized treatment. The continued translation of these advances into clinically applicable therapeutic strategies may help address the unmet therapeutic needs in CML, especially in the setting of advanced and blast-phase disease.

## 1. Introduction

Chronic myeloid leukemia (CML) represents a paradigmatic example of the successful implementation of targeted therapy in modern hematologic oncology. It is a prototypical BCR::ABL1-positive myeloproliferative neoplasm characterized by clonal expansion of predominantly granulocytic myeloid cells in the bone marrow and their accumulation in peripheral blood. The annual incidence of CML is approximately 1–2 cases per 100,000 individuals, accounting for approximately 15–20% of all adult leukemias [1,2].

The cytogenetic hallmark of CML is the Philadelphia chromosome (Ph), which is detected in nearly 95% of patients by conventional karyotyping. The same genetic abnormality is also found in approximately 20–30% of adult and 3–5% of pediatric patients with acute lymphoblastic leukemia (ALL) [3,4]. The Philadelphia chromosome most frequently originates from a reciprocal translocation between the long arms of chromosomes 9 and 22, t(9;22)(q34;q11), whereby the 5′region of the breakpoint cluster region (BCR) gene fuses with the 3′region of the Abelson proto-oncogene 1 (ABL1) gene. This chromosomal rearrangement gives rise to the *BCR::ABL1* fusion gene, which encodes a chimeric oncoprotein with constitutive tyrosine kinase activity [3,5] (Figure 1). In contrast to wild-type ABL1, whose kinase activity is tightly controlled by intramolecular autoinhibitory mechanisms, the BCR::ABL1 fusion protein lacks the ABL1 N-terminal myristoylation site required for physiological autoinhibition. Together with BCR-derived oligomerization, this disruption promotes persistent kinase activation and signaling that is largely independent of physiological regulatory cues [6,7]. Downstream, BCR::ABL1 signaling converges on the constitutive activation of multiple survival pathways, including RAS/MAPK, PI3K/AKT and JAK/STAT, ultimately establishing a proliferative and apoptosis-resistant leukemic phenotype that drives disease initiation and progression [8,9].

The clinical course of CML has traditionally been described as progressing through three consecutive phases—chronic phase, accelerated phase, and blast crisis, reflecting increasing genomic instability and a progressive decline in therapeutic responsiveness [10]. Prior to the advent of targeted therapy, treatment relied primarily on interferon-α, conventional cytotoxic chemotherapy, and allogeneic hematopoietic stem cell transplantation. These approaches provided limited disease control and were associated with an unfavorable long-term prognosis [11,12].

A major breakthrough in CML treatment came with the development of tyrosine kinase inhibitors (TKIs). The prototypical agent of this class, the small-molecule inhibitor imatinib, acts as a competitive inhibitor of BCR::ABL1 kinase activity and ushered in the era of TKIs as a cornerstone of targeted therapy in clinical hemato-oncology. Its introduction fundamentally altered the natural history of CML, markedly improving survival and transforming the disease into a chronically manageable condition for most patients [5,13,14].

The subsequent development of second-generation (dasatinib, nilotinib, and bosutinib) and the third-generation (ponatinib) TKIs substantially expanded the therapeutic options for CML, providing more potent inhibition of BCR::ABL1 and broader activity against resistant variants, including many kinase domain mutations. These agents have enabled faster and deeper molecular responses and improved clinical outcomes, including in patients who are intolerant of or resistant to first-line therapy [15,16]. More recently, asciminib has further expanded the therapeutic armamentarium as the first allosteric BCR::ABL1 inhibitor. By binding to the myristoyl pocket, asciminib stabilizes the kinase in an inactive conformation and restores the negative autoregulatory mechanism normally mediated by myristoyl binding, resulting in effective suppression of BCR::ABL1 kinase activity [17,18,19].

Despite these major therapeutic advances, primary and acquired TKI resistance remain important challenges in CML management, limiting durable disease control and contributing to the risk of disease relapse. A detailed understanding of BCR::ABL1 as a therapeutic target and of the molecular and cellular processes underlying therapeutic resistance is therefore essential for identifying actionable vulnerabilities and informing the development of more effective and individualized therapeutic approaches. Against this background, this narrative review provides an integrated overview of the molecular basis of TKI resistance and discusses current and emerging strategies for overcoming treatment failure and persistent disease.

## 2. BCR::ABL1 as a Therapeutic Target

BCR::ABL1 is the central oncogenic driver of CML and represents one of the most extensively validated pharmacological targets in oncology. The fusion protein combines regulatory and oligomerization domains derived from BCR with the tyrosine kinase domain of ABL1, resulting in constitutive kinase activity, autonomous proliferation, and impaired apoptotic signaling in leukemic cells [8,9]. 

Depending on the breakpoint within the BCR gene, distinct BCR::ABL1 transcripts and corresponding protein isoforms are generated. The predominant form in classical CML is p210 BCR::ABL1, encoded mainly by the e13a2 or e14a2 transcripts. Although both variants produce the p210 BCR::ABL1 protein of identical molecular weight, the e14a2 transcript has frequently been associated with an earlier and more rapid decline in BCR::ABL1 transcript levels and with deeper molecular responses to targeted therapy. Accordingly, transcript type may carry some prognostic value; however, it is not currently considered an independent criterion for TKI selection, which is guided primarily by disease phase, mutational profile, comorbidities, and prior treatment history [14,20].

As opposed to p210, the p190 BCR::ABL1 isoform, typically encoded by e1a2, predominates in Ph-positive B-cell ALL and has been associated with distinct protein–protein interactomes and differential activation profiles across the JAK/STAT, RAS/MAPK, PI3K/AKT/mTOR and Src family kinase pathways. It may also occur, albeit rarely, in CML, where it is generally associated with more aggressive clinicobiological features. p210 has been shown to preferentially engage STAT5- and ERK-mediated signaling, as well as STS1-dependent regulation of tyrosine kinase activity, whereas p190 preferentially recruits AP2/clathrin components and LYN, resulting in distinct signaling patterns that may contribute to their divergent leukemic phenotypes [21,22].

The p230 BCR::ABL1 is a considerably less common isoform and has been linked to more indolent myeloproliferative phenotypes, including neutrophilic variants of CML [8,23].

Despite their structural differences, all oncogenic BCR::ABL1 isoforms share the same ABL1 kinase domain. Consequently, direct binding of ATP-competitive TKIs is determined predominantly by the conformation and mutational status of this domain rather than by the size or molecular mass of the fusion protein. Nevertheless, experimental and clinical data suggest that the p190, p210, and p230 isoforms are associated with distinct leukemic phenotypes, pointing to the central role of the BCR-derived region in shaping disease biology independently of the shared ABL1 moiety [24,25]. This phenotypic diversity may be partly attributed to functional differences within the BCR-derived segment, which regulates oligomerization, subcellular localization and downstream signaling output and may consequently influence the depth and durability of therapeutic responses [26].

Conventional pharmacological targeting of BCR::ABL1 is therefore directed toward the conserved ABL1 kinase domain, which adopts a characteristic bilobal architecture, with the ATP-binding and catalytic sites located within the cleft between the N- and C-terminal lobes. This structural organization comprises several functionally important elements, including the phosphate-binding loop (P-loop), hinge region, catalytic loop (C-loop), activation loop (A-loop), and the highly conserved HRD and DFG motifs. Coordinated conformational rearrangements within these regions determine whether the kinase domain adopts an active or inactive state and, consequently, which classes of inhibitors can bind to it most effectively [27,28,29].

The catalytic activity of the native ABL1 kinase is tightly controlled by autoregulatory mechanisms involving a network of intramolecular interactions. Docking of the myristoylated N terminus, present in the ABL1b splice variant, into a hydrophobic pocket within the C-terminal lobe, coupled with contacts between regulatory regions and the SH3 and SH2 domains, generates allosteric inhibitory signals that reshape the kinase and stabilize a compact inactive conformation. This tightly controlled autoinhibitory mechanism is disrupted upon the formation of the fusion BCR::ABL1 oncoprotein, as replacement of the native ABL1 N-terminal regulatory region by BCR-derived sequences and BCR-mediated oligomerization favor persistent kinase activation and stabilize the enzyme in a constitutively active state [30,31].

From a pharmacological perspective, the dynamic equilibrium between the active DFG-in and inactive DFG-out conformations is particularly important. Imatinib and nilotinib (type II inhibitors) preferentially bind and stabilize the inactive DFG-out conformation of ABL1, whereas dasatinib (type I inhibitor) is less constrained by the conformational state of the kinase and can bind both active and inactive conformations. These differences contribute to their distinct activity spectra, target selectivity and susceptibility to specific kinase domain mutations [32].

Elucidation of the endogenous mechanisms governing negative regulation of ABL1 kinase activity provided the conceptual basis for a new therapeutic strategy aimed at mimicking the physiological allosteric effects of N-terminal myristoylation. The development of asciminib as the prototypical agent of this class expanded the pharmacological targeting of BCR::ABL1 beyond the ATP-binding pocket and enabled combination strategies based on simultaneous inhibition of the catalytic and allosteric regulatory sites [19,33,34]. A detailed understanding of the structural and functional properties of BCR::ABL1 therefore provides the foundation for the development of next-generation ATP-competitive and allosteric inhibitors, as well as rational combination strategies designed to overcome therapeutic resistance.

## 3. Clinically Established TKIs

The development and clinical introduction of tyrosine kinase inhibitors (TKIs) represent one of the most significant achievements of modern rational medicinal chemistry and marked the beginning of the era of small-molecule targeted therapy in various types of malignancies. Advances in understanding the structure of BCR::ABL1 and the mechanisms governing its tyrosine kinase activity provided the foundation for the development of drug inhibitors capable of selectively targeting the ATP-binding pocket of the enzyme. The emergence of the concept of competitive orthosteric inhibition of tyrosine kinases by small molecules, exemplified by the tyrphostins, together with rational drug-design efforts aimed at improving target selectivity, ultimately led to the clinical introduction of imatinib in 2001 as the prototypical agent of a new class of BCR::ABL1-targeted therapeutics [35] (Figure 2).

### 3.1. ATP-Competitive TKIs

Imatinib is a pyrimidine derivative that acts through an ATP-competitive mechanism and, in addition to BCR::ABL1 and ABL1, inhibits several other oncogenic tyrosine kinases, including c-KIT, ARG (ABL2), and platelet-derived growth factor receptors (PDGFRs). Aberrant activation of c-KIT (CD117) and PDGFRA has a well-established pathogenic role in gastrointestinal stromal tumors (GISTs), prompting the additional approval of imatinib for the treatment of patients with advanced GIST [36,37].

Long-term results from the eight-year follow-up of the IRIS trial demonstrated that imatinib treatment resulted in complete cytogenetic response in approximately 83% of patients, with overall survival reaching approximately 85% [38]. Despite these remarkable outcomes, early clinical studies revealed that a subset of patients, particularly those with accelerated-phase or blast-phase disease, developed resistance followed by disease relapse [38,39]. Subsequent molecular studies established BCR::ABL1 kinase-domain mutations as a major mechanism of acquired TKI resistance, examined in detail in Section 4.1.

Nilotinib is a structural analogue of imatinib in which rational molecular modifications improve complementarity with the inactive DFG-out conformation of ABL1, resulting in higher binding affinity and approximately 30-fold greater inhibitory potency against wild-type BCR::ABL1 in preclinical models [40]. This optimized interaction also preserves activity against most imatinib-resistant BCR::ABL1 variants, with the notable exception of T315I. Nilotinib was initially established as second-line therapy for patients with CML who were resistant or intolerant to imatinib and was subsequently introduced as a first-line treatment option for newly diagnosed Ph-positive CML in chronic phase [41,42]. 

Dasatinib represents a structurally distinct ATP-site inhibitor incorporating a thiazole-carboxamide pharmacophore that enables binding to both active and inactive conformations of ABL1 and extends inhibitory activity to SRC-family kinases, including Lyn [32,43,44]. This broader conformational and kinase-binding profile contributes to its approximately 300-fold greater potency than imatinib against wild-type BCR::ABL1 and activity against many imatinib-resistant kinase-domain mutations [43,45]. Inhibition of SRC-family kinases is also relevant to BCR::ABL1-independent resistance, as persistent Lyn/SRC signaling can provide an alternative route for maintaining leukemic-cell survival. Dasatinib may additionally circumvent certain mechanisms of multidrug resistance, as its interactions with P-glycoprotein differ from those of imatinib [44]. Its broader kinase inhibitory activity, however, can also affect immune-cell signaling and interfere with CD8^+^ T-cell function and dendritic-cell activation [46,47]. Moreover, inhibition of SRC-family and other off-target kinases contributes to the characteristic safety profile of the drug, including cardiovascular toxicity, that constitute important considerations in its clinical use [48].

The development of ponatinib marked a major turning point in the evolution of TKIs. Ponatinib was generated through structure-based rational drug design specifically to overcome the T315I “gatekeeper” mutation [15]. Incorporation of a linear ethynyl (C≡C) linker into its molecular scaffold circumvents the steric hindrance imposed by the bulky isoleucine residue at position 315, thereby enabling effective accommodation within the ATP-binding site. Consequently, ponatinib retains potent activity against T315I and nearly all clinically relevant single BCR::ABL1 kinase domain mutations and is therefore commonly regarded as a pan-BCR::ABL1 inhibitor [16,49]. Its kinase-inhibition spectrum also extends to SRC-family kinases, PDGFRα, c-KIT, and FLT3, providing a pharmacological rationale for its investigation in other molecularly defined hematologic malignancies, including FLT3-mutated AML [50,51]. The clinical scope of ponatinib was further expanded in March 2024, when the FDA granted accelerated approval for its use in combination with chemotherapy in adults with newly diagnosed Ph-positive ALL, based on the results from the phase III PhALLCON trial (NCT03589326) [52].

As with many TKIs, ponatinib has also been associated with clinically important cardiovascular toxicity; however, it has been reported to exert immunomodulatory effects distinct from those of dasatinib, including promotion of CD8^+^ T memory stem cell (TSCM) differentiation and enhancement of antitumor immune responses [50]. The benefit-risk profile of ponatinib has subsequently been refined through response-adapted dosing. In the prospective phase II OPTIC trial, the optimal benefit-risk balance was observed with a starting dose of 45 mg once daily followed by reduction to 15 mg upon achievement of BCR::ABL1IS ≤1%, providing a strategy to preserve efficacy while reducing dose-dependent toxicity [53].

### 3.2. Allosteric BCR::ABL1 Inhibitors

A more detailed understanding of the structural and regulatory mechanisms governing ABL1 activity enabled a fundamentally different approach based on allosteric rather than ATP-competitive inhibition. Asciminib selectively binds the myristoyl pocket of BCR::ABL1, stabilizing an inactive kinase conformation independently of the orthosteric ATP-binding site. Reflecting this mechanism, the term STAMP (Specifically Targeting the ABL Myristoyl Pocket) was introduced to distinguish asciminib from conventional ATP-competitive TKIs [54].

This structurally and mechanistically distinct mode of inhibition avoids direct competition at the highly conserved ATP-binding site, providing greater target selectivity and reducing much of the off-target kinase activity associated with broader ATP-site inhibition. Importantly, occupation of a spatially distinct regulatory pocket also enables activity against problematic BCR::ABL1 kinase domain mutations, including T315I, although effective inhibition may require higher drug exposure. This expands the potential therapeutic options for patients with CML and other Ph^+^ malignancies harboring resistant mutations, as well as for those in whom long-term ponatinib treatment may be limited by the risk of serious cardiovascular events [18,33,34].

In October 2021, the FDA granted accelerated approval to asciminib for the treatment of patients with CML who had previously received at least two lines of TKI therapy or who harbored the T315I mutation [17,18]. This indication was subsequently expanded in October 2024, when the FDA granted accelerated approval to asciminib for adults with newly diagnosed Ph-positive CML in chronic phase, establishing its role as a frontline therapeutic option. The approval was supported by the phase III ASC4FIRST trial (NCT04971226), in which asciminib achieved a significantly higher MMR rate at week 48 than investigator-selected TKIs (67.7% vs. 49.0%) [55,56].

## 4. Molecular Mechanisms of Resistance in CML

The development of TKIs has fundamentally transformed the prognosis of patients with CML; however, therapeutic resistance remains a major obstacle to durable disease control. Clinically, resistance may be primary (de novo), defined by failure to achieve the expected hematologic, cytogenetic or molecular treatment milestones, or acquired, characterized by loss of a previously established response during therapy [57,58,59].

At the molecular level, TKI resistance can be broadly categorized as BCR::ABL1-dependent or BCR::ABL1-independent (Figure 3) [60]. The former results from structural or quantitative alterations of BCR::ABL1 that impair effective target inhibition, whereas the latter encompasses cell-intrinsic and microenvironmental adaptations that sustain leukemic cell survival despite suppression of BCR::ABL1 signaling [60,61].

Importantly, these mechanisms do not occur as isolated events and may coexist or evolve under therapeutic pressure. Their interplay, involving genetic alterations, adaptive signaling, microenvironmental protection, among others, contributes to the molecular heterogeneity of TKI resistance and to differential responses across TKI generations [62]. This complexity underscores the importance of molecular profiling that extends beyond BCR::ABL1 mutation analysis to capture the broader molecular landscape of TKI resistance and inform individualized treatment decisions.

### 4.1. BCR::ABL1-Dependent Mechanisms of Resistance

BCR::ABL1-dependent mechanisms represent the most extensively characterized group of molecular events underlying drug resistance in CML. Their common phenotypic manifestation is the direct alteration of the BCR::ABL1 oncoprotein, which interferes with effective TKI binding and enables restoration or persistence of aberrant tyrosine kinase signaling. These mechanisms predominantly involve point mutations within the kinase domain but also include BCR::ABL1 gene amplification and overexpression, aberrant splicing, and less frequent structural alterations outside the ATP-binding region, including mutations affecting the myristoyl-binding pocket [62,63,64]. 

The development of BCR::ABL1-dependent resistance is a dynamic adaptive process driven by the selective pressure imposed by prolonged TKI therapy. During treatment, TKI-sensitive cell populations are progressively eliminated, whereas pre-existing or newly emerging mutant subclones gain a selective advantage and may eventually dominate the leukemic population. This pattern of clonal evolution has been well documented in both clinical and experimental studies and represents a central concept in the contemporary understanding of drug resistance in CML [65,66]. Importantly, resistance-associated mutations do not necessarily occur as isolated genetic events. Under the combined pressure of prolonged therapy and the intrinsic genomic instability of leukemic cells, additional mutations may emerge and multiple alterations may accumulate sequentially within the same BCR::ABL1 allele. This process can give rise to complex resistant subclones with distinct molecular characteristics, further complicating therapeutic management and contributing to disease progression toward accelerated phase or blast crisis [67,68].

#### 4.1.1. BCR::ABL1 Mutational Profiling and Its Clinical Relevance 

Accordingly, BCR::ABL1 kinase domain mutation analysis has become an integral component of molecular monitoring in CML, particularly in patients experiencing treatment failure or loss of molecular response. Current European LeukemiaNet recommendations emphasize mutational profiling when a change in TKI therapy is considered, as it is instrumental in guiding the choice of appropriate next-line treatments and may provide additional prognostic information [69]. 

A major advance in the molecular characterization of resistance has been the implementation of next-generation sequencing (NGS), which enables more accurate discrimination between compound and polyclonal mutations. Although Sanger sequencing can identify individual amino acid substitutions, it generally cannot establish whether multiple mutations reside within the same BCR::ABL1 transcript or occur in distinct leukemic subclones [70]. Long-read NGS approaches enable mutation phasing and characterization of clonal heterogeneity within leukemic populations, substantially improving the resolution with which resistant clones can be defined. This distinction has important clinical implications, as co-occurring mutations within the same BCR::ABL1 allele are frequently associated with a lower probability of achieving a durable molecular response and may warrant earlier consideration of alternative therapeutic strategies [71]. 

In practical terms, BCR::ABL1 kinase-domain mutation profiling is recommended in patients with treatment failure or warning responses, loss of MMR, or disease progression, whereas routine testing at diagnosis or in patients with BCR::ABL1^IS < 0.1% is generally not indicated. Targeted NGS is preferred when available because its greater sensitivity enables earlier detection of emerging resistant clones and may facilitate identification of compound mutations; Sanger sequencing remains an acceptable alternative where NGS is not accessible. Mutation analysis should be repeated when the response fails to improve or subsequently worsens after a TKI switch, while low-level variants of uncertain significance may warrant reassessment after 1–3 months [69,72]. Importantly, the mutation profile directly informs treatment selection by excluding TKIs with reduced activity against the detected variant. Compound mutations require special consideration because their sensitivity profile may differ substantially from that of the corresponding individual mutations and may confer resistance even to ponatinib or asciminib, particularly when T315I is involved. In these cases, early consideration of allo-HSCT is warranted [69].

#### 4.1.2. Mutations in the BCR::ABL1 Kinase Domain

As outlined in Section 2, the structural organization and conformational dynamics of the BCR::ABL1 kinase domain critically determine TKI binding. Accordingly, mutations affecting its key functional regions can differentially impair inhibitor recognition and activity.

Point mutations within the BCR::ABL1 kinase domain represent the most frequent and extensively studied mechanisms of acquired TKI resistance. They are usually detected in patients who have lost a previously achieved molecular or cytogenetic response, and constitute a major cause of treatment failure with earlier-generation ATP-competitive inhibitors [65,66]. To date, more than 100 distinct mutations affecting over 50 amino acid residues across different regions of the kinase domain have been identified. Their prevalence, functional consequences, and clinical significance vary considerably depending on both their structural location and the specific amino acid substitution involved [73]. The major groups of BCR::ABL1 kinase domain mutations and their key characteristics are summarized in Table 1.

**Table 1:** Key mutations within the structural and functional regions of the BCR::ABL1 kinase domain and their impact on TKI sensitivity and resistance.

Structural-Functional Segment	Primary Function	Common Mutations	Clinical Relevance	Ref.
**P-loop (phosphate-binding loop)**	Binds and stabilizes ATP; contributes to substrate positioning.	G250E, Q252H/R, Y253F/H, E255K/V	Common mutations associated with high-level resistance to imatinib; most also confer reduced sensitivity to second-generation TKIs.	[74,75,76,77]
**Gatekeeper residue (Thr315)**	Controls access to the ATP-binding pocket and forms a critical hydrogen bond with ATP-competitive TKIs.	**T315I**	The most clinically significant mutation; confers resistance to all ATP-competitive TKIs except ponatinib. Asciminib generally retains activity against isolated T315I mutations; however, its efficacy may be compromised in the presence of T315I compound mutations.	[78,79,80,81,82]
**Hinge region**	Links the N- and C-lobes and contributes to interactions with ATP and TKIs.	F317L/V/I/C	Most commonly associated with resistance to imatinib and dasatinib, while sensitivity to the other second-generation TKIs (nilotinib and bosutinib) is generally better preserved.	[9,76,77,83,84,85]
**Catalytic loop (C-loop)**	Contains the HRD motif and contributes to the catalytic activity of the kinase.	M351T, F359V/C/I	M351T generally confers moderate-to-high levels of resistance to imatinib, while usually retaining sensitivity to second-generation TKIs and ponatinib. Bosutinib and dasatinib are preferred therapeutic options for F359V/C/I mutations.	[62,86,87]
**Activation loop (A-loop)**	Regulates the transition between the active (DFG-in) and inactive (DFG-out) conformations	H396R/P	Stabilizes the active kinase conformation; primarily associated with resistance to imatinib, while sensitivity to second-generation TKIs is generally preserved.	[84,88,89,90]

- The T315I Gatekeeper Mutation

Among mutations affecting the BCR::ABL1 kinase domain, the T315I gatekeeper mutation, in which threonine at position 315 is replaced by isoleucine, is of greatest clinical significance. This amino acid substitution affects a critical residue within the ATP-binding pocket and represents a prototypical example of structurally mediated TKI resistance [82,91]. 

The molecular consequences of T315I are twofold. First, loss of the threonine -OH group eliminates a critical H-bond required for stable binding of imatinib and other ATP-competitive TKIs. Second, the bulkier and more hydrophobic isoleucine side chain introduces steric hindrance within the ATP-binding pocket, thereby preventing the optimal positioning of inhibitor molecules [92,93]. Consequently, T315I confers broad cross-resistance to first- and second-generation TKIs, including imatinib, nilotinib, dasatinib, and bosutinib [76]. Its therapeutic impact is further amplified when it occurs within compound BCR::ABL1 variants, as discussed below.

The impact of T315I extends beyond direct impairment of drug binding and also involves alterations in the conformational equilibrium of the BCR::ABL1 kinase domain. The mutation can favor structural states that are less efficiently recognized by inhibitors with specific conformational requirements, including those dependent on a particular DFG orientation, further compromising effective target inhibition [78,91,94]. In this context, the development of ponatinib represented a major therapeutic breakthrough for T315I-mediated resistance. The structure-guided rationale for its design is discussed in Section 5.1.

The clinical efficacy of ponatinib in patients with resistant CML, including those harboring T315I, was demonstrated in the phase II PACE trial, in which more than 90% of patients had previously received at least two TKIs. Among patients with chronic-phase CML, 56% achieved a major cytogenetic response, while the 12-month progression-free survival (PFS) and overall survival (OS) rates were 80% and 94%, respectively. The pivotal findings from this study established ponatinib as a third-generation TKI and an important therapeutic option for patients with T315I-positive and multidrug-resistant CML [95]. 

More recently, asciminib has provided an alternative therapeutic strategy for patients with T315I-positive CML. As the first clinically validated and approved STAMP (Specifically Targeting the ABL Myristoyl Pocket) inhibitor, asciminib binds to the ABL1 myristoyl pocket rather than the ATP-binding site. Owing to this distinct allosteric mechanism, asciminib retains activity in a subset of patients with T315I-positive CML, including patients with prior ponatinib failure. In a phase I study involving heavily pretreated patients with CML, major molecular response (MMR) was achieved or maintained by 12 months in 48% of patients with chronic-phase disease, including 57% of those refractory to ponatinib treatment. Among patients harboring T315I, MMR was achieved in 28%, providing clinical evidence that allosteric targeting can bypass the structural constraints imposed by this mutation on conventional ATP-competitive inhibition [19,96]. Notably, asciminib activity against T315I is dose-dependent, with a substantially higher recommended dose (200 mg twice daily) than that used in patients with CML-CP without the T315I mutation [97].

- P-loop mutations

In addition to T315I, mutations within the phosphate-binding loop (P-loop) represent one of the most frequent groups of BCR::ABL1 mutations detected in patients with acquired TKI resistance. They account for approximately 35–45% of kinase domain mutations and affect the flexible ATP-binding region involved in the correct positioning of ATP during the catalytic cycle [74,98]. This region plays a critical role not only in kinase activity but also in stabilizing the inactive DFG-out conformation of BCR::ABL1 required for efficient imatinib binding. Consequently, structural alterations within the P-loop can simultaneously impair inhibitor binding and enhance kinase catalytic activity, resulting in varying degrees of resistance to first- and second-generation TKIs, while generally retaining sensitivity to ponatinib [99]. 

The most frequently reported mutations within this region include G250E, Q252H, Y253F/H, and E255K/V, each of which exerts distinct effects on the topology of the ATP-binding pocket and on the sensitivity of leukemic cells to different generations of TKIs [93]. 

Among P-loop alterations, E255K and E255V are of particular clinical relevance. Substitution of glutamate by lysine or valine substantially alters the electrostatic environment of the ATP-binding pocket and weakens the interaction between BCR::ABL1 and several ATP-competitive inhibitors. Beyond these structural effects, E255K/V may increase the catalytic activity of the kinase, further enhancing the proliferative capacity of leukemic cells. Clinical observations have associated these mutations with an increased risk of progression to accelerated-phase disease and shorter progression-free survival compared with many other single kinase domain mutations [100,101]. 

The G250E mutation introduces a negatively charged glutamate residue into a highly flexible region, altering the local conformation of the P-loop and weakening the interaction with imatinib. Although the degree of resistance is generally lower than that associated with T315I or E255K/V, G250E may also be associated with a suboptimal molecular response to second-generation TKIs, potentially warranting adjustment of the therapeutic strategy or dosing regimen [102,103]. 

The Q252H mutation alters the spatial arrangement of neighboring amino acid residues and affects the structural stability of the ATP-binding region. Clinically, this mutation is most commonly associated with reduced sensitivity to imatinib. Despite evidence of reduced dasatinib sensitivity in some in vitro studies, clinical responsiveness generally remains preserved [102,104].

Of considerable clinical significance for resistance to older-generation TKIs are Y253F and Y253H, which affect a residue involved in P-loop stabilization. Substitution of Tyr253 with phenylalanine or histidine perturbs the local network of molecular interactions and destabilizes the inactive DFG-out conformation, substantially reducing the affinity of imatinib and nilotinib for BCR::ABL1, whereas dasatinib and bosutinib may retain activity against these variants [102,105].

- Mutations in the C-loop and A-loop

In addition to alterations affecting the ATP-binding region, mutations within the catalytic loop (C-loop) and activation loop (A-loop) have also been reported. Unlike T315I, most mutations within these regions are less frequent and do not confer broad cross-resistance across all TKI generations. Their clinical significance depends on the specific amino acid substitution, the TKI of choice, and the presence of additional co-occurring mutations [106]. 

The C-loop contains the highly conserved HRD motif, which participates directly in the catalytic transfer of a phosphoryl group from ATP to the substrate. Mutations within this region can alter the spatial organization of the catalytic center, affect the efficiency of ATP-dependent catalysis, and modify the stability of the enzyme-substrate complex [107]. Their clinical impact, however, is heterogeneous. M351T is associated with a several-fold increase in the IC_50_ of imatinib relative to wild-type BCR::ABL1, while sensitivity to later-generation TKIs is generally preserved. In contrast, F359V/C/I mutations show reduced sensitivity to imatinib and nilotinib but retain greater sensitivity to dasatinib and bosutinib, making the latter agents preferred therapeutic options in these cases [62,86,87]. 

Mutations within the A-loop of BCR::ABL1 are of particular interest because this region functions as a molecular switch governing the transition between the active and inactive conformations of the kinase center. Under physiological conditions, the equilibrium between these states is tightly regulated, ensuring precise control of catalytic activity. A-loop mutations can disrupt this conformational equilibrium of the kinase domain, stabilizing catalytically active conformations and reducing its propensity to adopt the inactive DFG-out state required for optimal binding of type II TKIs such as imatinib and nilotinib [63]. 

Among A-loop mutations, H396P is a well-characterized example, in which substitution of histidine by proline induces local conformational rigidity within the A-loop. This structural alteration impairs the transition toward the inactive conformation and favors maintenance of the kinase in a catalytically active state. Consequently, H396P is associated with reduced sensitivity to imatinib, whereas sensitivity to newer-generation TKIs is generally preserved [84,88,89,90].

- Compound mutations

The accumulation of two or more BCR::ABL1 mutations adds another layer of complexity in the molecular evolution of drug resistance in CML. Depending on the distribution of the individual alterations, compound and polyclonal mutations can be distinguished, representing two phenomena with distinct biological and clinical implications.

In compound mutations, two or more amino acid substitutions occur within the same BCR::ABL1 allele and are therefore expressed within the same protein molecule. In contrast, polyclonal mutations arise in distinct leukemic subclones, with each cell population harboring a different mutant variant of BCR::ABL1 transcripts. Although both forms may coexist in the same patient, compound mutations are generally associated with higher levels of drug resistance and a less favorable prognosis [108,109]. Their emergence frequently reflects clonal evolution of leukemic cells under the selective pressure imposed by successive TKI therapies. Moreover, their clinical relevance is likely to increase as improved patient survival extends disease courses and leads to sequential exposure to multiple lines of targeted therapy.

The functional consequences of compound mutations may substantially exceed those of the corresponding individual mutations. The presence of two or more amino acid substitutions within the same BCR::ABL1 molecule can induce extensive remodeling of the three-dimensional organization of the kinase domain, simultaneously altering the geometry of the ATP-binding pocket, the local electrostatic environment, the conformational equilibrium between the DFG-in and DFG-out states, and the kinetics of inhibitor binding [68,110]. Consequently, TKI efficacy may be markedly reduced, reflecting a greater-than-additive loss in inhibitor binding affinity. The most clinically challenging compound BCR::ABL1 variants are those harboring T315I, which may remain refractory even to the most potent TKIs specifically developed to overcome T315I-mediated resistance [111]. 

A representative example is T315I/E255V, in which the combination of the gatekeeper T315I substitution with the P-loop E255V alteration confers broad cross-resistance to ATP-competitive TKIs. Whereas ponatinib effectively inhibits BCR::ABL1 harboring T315I as a single mutation, the additional presence of E255V markedly reduces its inhibitory activity against the kinase. Similar patterns of cross-resistance have been reported for T315I/Y253H, T315I/F317L, and T315I/H396R, which exhibit varying degrees of sensitivity to ponatinib while showing profound resistance to other ATP-competitive inhibitors. Compound mutations not involving T315I, including Y253H/E255V, E255V/F317I, and E255K/F317L, may likewise confer reduced, although variable, sensitivity to different generations of TKIs [112].

#### 4.1.3. BCR::ABL1 Mutations Beyond the Kinase Domain

Although most clinically relevant mutations affect the BCR::ABL1 kinase domain, available experimental and clinical evidence indicates that structural alterations outside the ATP-binding pocket can also modulate kinase activity and TKI sensitivity. Unlike mutations within the catalytic domain, alterations affecting regulatory regions generally do not directly interfere with TKI binding. Instead, they modify the three-dimensional organization and conformational dynamics of the kinase, and can impair the TKI recognition and binding affinity [113].

- Mutations in highly conserved N-terminal regions

The best-characterized genetic alterations outside the ABL1 kinase region are point mutations within the SH2 and SH3 domains, highly conserved N-terminal regulatory regions located immediately upstream of the catalytic unit. The SH3 domain recognizes proline-rich motifs and contributes to stabilization of the inactive kinase state, whereas the SH2 domain recognizes phosphotyrosine-containing motifs and interacts with the kinase center. Together with the SH2-kinase linker and the N-terminal myristoylated region, these intramolecular interactions maintain the autoinhibitory conformation of ABL1 and provide tight control over its tyrosine kinase activity [113,114].

Experimental models suggest that mutations within these regulatory units can favor or “lock” BCR::ABL1 in an active conformation, resulting in increased catalytic activity, enhanced autophosphorylation, and activation of downstream signaling pathways. Based on their predicted conformational effects, these alterations could reduce the efficacy of TKIs that preferentially bind the inactive DFG-out state (type II TKIs), including imatinib and nilotinib. Limited clinical evidence for dasatinib, which can bind the kinase in both active and inactive conformational states, suggests that its activity may be less affected [115].

- Mutations in the Myristoyl-binding pocket

The introduction of asciminib into clinical practice has broadened the understanding of BCR::ABL1-dependent resistance by imposing a new selective pressure on the allosteric myristoyl-binding pocket of ABL1. Unlike ATP-competitive TKIs, asciminib binds outside the ATP-binding site and stabilizes the autoinhibited conformation of the kinase, raising the probability for a distinct form of resistance driven by structural alterations within the regulatory myristoyl pocket or adjacent regions [33,34]. 

Mutations arising within this allosteric region can compromise the activity of asciminib through several mechanisms, including direct disruption of molecular interactions between the drug and its binding site, alterations in the local hydrophobic geometry of the pocket or impairment of allosteric communication between the myristoyl pocket and the kinase domain. Among the most frequently discussed variants are A337V, P223S, C464W, P465S, V468F, and I502L, some of which have been identified in preclinical selection models and others in patients who developed resistance or lost response to asciminib therapy [96,116,117]. A viable therapeutic approach to mitigate the selective pressure driving resistant clonal expansion is the so-called dual-site inhibition, in which BCR::ABL1 is simultaneously targeted at the ATP-binding site and the myristoyl pocket. Combining asciminib with an ATP-competitive TKI may reduce the likelihood of resistant-clone selection, as complete escape from pharmacological inhibition would require concurrent adaptive alterations affecting two functionally distinct binding sites within BCR::ABL1 [33].

The clinical implications of these mutations differ from those of classical kinase domain mutations. Whereas T315I confers cross-resistance to most ATP-competitive TKIs, isolated myristoyl-pocket mutations predominantly affect the pharmacological activity of asciminib and, in the absence of additional resistance-associated mechanisms, do not necessarily reduce sensitivity to ponatinib, dasatinib, nilotinib, or bosutinib. Conversely, asciminib can retain inhibitory activity against certain mutations within the ATP-binding region because its binding does not depend on direct interactions with this site. Consistent with this concept, in the early-phase CABL001X2101 clinical trial, asciminib demonstrated good clinical efficacy in patients with chronic-phase CML, including a subgroup harboring the T315I mutation and other difficult-to-treat types of resistance to ATP-competitive TKIs [96].

#### 4.1.4. Alternative Splicing of BCR::ABL1

In addition to genetic alterations at the DNA level, BCR::ABL1-dependent resistance may also be influenced by dysregulation of the post-transcriptional processing of the pre-mRNA encoding the fusion protein. Advances in RNA-sequencing technologies have revealed that the transcriptomic diversity of BCR::ABL1 is considerably greater than initially recognized. Multiple alternative transcripts have been identified, although the functional significance of most remains poorly understood. Whether some of these variants influence BCR::ABL1 stability, intracellular localization, or interactions with different classes of TKIs remains to be established [118,119,120]. 

The best-characterized example is the BCR::ABL1^35INS splice variant, which results from the insertion of 35 nucleotides derived from the intronic sequence between exons 8 and 9 of ABL1. This variant was initially identified in patients who had lost molecular response to imatinib and was therefore proposed as a novel mechanism of acquired resistance [118]. Subsequent functional studies, however, have challenged this hypothesis. The resulting truncated protein has been shown to exhibit markedly reduced or absent tyrosine kinase activity and is unable to independently sustain leukemogenic signaling [120]. These findings suggest that BCR::ABL1^35INS is unlikely to represent a primary molecular driver of resistance and may instead reflect aberrant post-transcriptional processing occurring in the context of other resistance-associated alterations. Clinically, this splice variant has been detected both in patients with imatinib resistance and in those treated with second-generation TKIs, including dasatinib and nilotinib. To date, however, no convincing causal relationship has been established between the presence of BCR::ABL1^35INS and reduced sensitivity to any specific TKI [120,121,122].

#### 4.1.5. BCR::ABL1 Gene Amplification and Overexpression

In addition to structural alterations, another well-established BCR::ABL1-dependent mechanism of resistance is increased BCR::ABL1 expression, which may result from gene amplification, an increased number of Philadelphia chromosome copies, or dysregulation of transcriptional control of the fusion oncogene [123,124]. Importantly, BCR::ABL1 gene amplification should be distinguished from an increase in BCR::ABL1 transcript levels detected during routine molecular monitoring. An increase in BCR::ABL1^IS may reflect loss of molecular response, poor treatment adherence, pharmacokinetic factors, expansion of a resistant subclone, or a true increase in oncogene copy number. Accordingly, confirmation of gene amplification requires additional analyses, such as fluorescence in situ hybridization (FISH), quantitative PCR, cytogenetic analysis, or other copy-number assessment methods [69]. 

BCR::ABL1 gene amplification was identified as one of the earliest mechanisms of imatinib-induced resistance in experimental models, and its emergence has been associated with increased genomic instability [125]. At high levels of oncogene expression, the intracellular bioavailability of the drug may be insufficient to inhibit the entire pool of BCR::ABL1 molecules, allowing persistent phosphorylation of critical substrates and sustained activation of downstream proliferative signaling pathways [123,124]. Because increased BCR::ABL1 abundance may exceed the pharmacological coverage achieved with less potent inhibitors such as imatinib, dose escalation generally provides limited benefit, potentially necessitating a switch to more potent second- or third-generation TKIs [126]. 

An intriguing apparent paradox is that the theoretical frequency of BCR::ABL1 gene amplification has been estimated to be substantially higher (10^−4^ per cell division) than that of point mutations (10^−9^ per cell division), yet amplification is detected less frequently in patients and, in some cases, coexists with kinase domain point mutations. One proposed explanation is that excessive BCR::ABL1 expression may itself impose a fitness disadvantage on leukemic cells. Indeed, leukemic cells overexpressing BCR::ABL1 may lose viability and proliferative capacity following imatinib withdrawal, a phenomenon attributed to alterations in their biological properties, including growth factor dependence, clonogenic potential and migratory capacity. Moreover, such cell populations can generate resistant mutant subclones more rapidly, suggesting that BCR::ABL1 overexpression may accelerate clonal evolution. Given that BCR::ABL1 transcript levels correlate with disease phase, this mechanism may contribute to the more rapid emergence of resistance during accelerated phase and blast crisis [127,128].

### 4.2. BCR::ABL1-Independent Mechanisms of Resistance

Unlike BCR::ABL1-dependent resistance, which arises through alterations directly affecting the molecular target, BCR::ABL1-independent resistance encompasses a diverse range of processes involving cellular signaling, drug transport and metabolism, interactions with the bone marrow microenvironment and the biology of LSCs. Rather than acting in isolation, these processes converge within an adaptive signaling circuitry that enables small LSC populations to survive, promotes the persistence of minimal residual disease and facilitates the gradual emergence of clinical resistance during TKI therapy. Moreover, persistent LSCs are considered a major cellular reservoir underlying late disease relapse, including relapse following TKI discontinuation [8,60,93]. A deeper understanding of these pathobiological features is therefore essential for the development of novel multimodal therapeutic strategies based on rational drug combinations and the simultaneous targeting of multiple cellular and immunological mechanisms involved in CML progression.

#### 4.2.1. Impaired Intracellular Transport of TKIs

The molecular target of TKIs is the cytosolic non-receptor tyrosine kinase BCR::ABL1; consequently, their therapeutic efficacy is highly dependent on their subcellular distribution and their ability to achieve and maintain adequate intracellular concentrations within leukemic cells.

Accordingly, interactions with membrane transport systems governing the cellular influx and efflux of drug molecules represent an important component of BCR::ABL1-independent resistance. Alterations in the expression or activity of these transporters can reduce intracellular drug exposure despite adequate plasma concentrations, resulting in suboptimal target coverage and facilitating the emergence and persistence of leukemic resistance [63,127]. However, the clinical relevance of membrane transporters and their utility as independent predictors of therapeutic response remain a matter of debate. Their contribution is likely to be most pronounced during the early stages of treatment and in patients without BCR::ABL1 mutations, whereas in the setting of advanced clonal evolution, transporter-mediated effects generally coexist with other resistance mechanisms [129,130]. Nevertheless, intracellular TKI transport represents an important pharmacological determinant of interindividual variability in therapeutic response and should be considered an integral component of the complex mechanistic repertoire driving BCR::ABL1-independent resistance.

The best-characterized transporter implicated in the active uptake of TKIs into leukemic cells is the organic cation transporter 1 (OCT1; SLC22A1), which transports a broad range of organic cations, including imatinib. Reduced OCT1 expression or functional activity limits intracellular accumulation of imatinib in leukemic cells and has been associated with lower rates of MMR and an increased risk of treatment failure [127,130]. Clinical studies have suggested that patients with low OCT1 activity exhibit shorter progression-free survival and may more frequently require imatinib dose escalation or switching to a second-generation TKI [131,132].

Importantly, the contribution of OCT1 to cellular uptake differs substantially among TKIs. For example, imatinib internalisation has been widely regarded as OCT1-dependent, whereas dasatinib, nilotinib, bosutinib and ponatinib enter cells predominantly through passive diffusion or alternative transport mechanisms. Consequently, reduced OCT1 activity appears to be of considerably less clinical relevance during treatment with second- and third-generation TKIs [133,134].

In contrast to OCT1, members of the ATP-binding cassette (ABC) transporter family function as energy-dependent efflux pumps that reduce the intracellular concentrations of a wide range of structurally diverse xenobiotics. In CML, ABCB1 (P-glycoprotein; MDR1) and ABCG2 (breast cancer resistance protein; BCRP) are of particular relevance [127]. ABCB1 is one of the best-characterized mediators of multidrug resistance in oncology. Its overexpression enhances the active efflux of imatinib and may result in subtherapeutic intracellular drug concentrations despite adequate plasma exposure. Accumulating experimental and clinical evidence suggests that imatinib is particularly susceptible to ABCB1-mediated resistance, whereas the more potent TKIs dasatinib and ponatinib appear to be less affected by this mechanism [134,135,136]. 

ABCG2 represents another major efflux transporter involved in regulating intracellular TKI exposure. Evidence from multiple experimental studies indicates that increased ABCG2 expression can reduce the intracellular availability of imatinib, dasatinib, and nilotinib, whereas ponatinib appears to be minimally affected [130,135,136,137,138].

The relatively limited dependence of ponatinib on membrane transport systems is noteworthy. This behavior may be partly attributed to its distinctive structural and physicochemical profile, including a rigid central ethynyl linker, a highly lipophilic −CF_3_ substituent, and overall high lipophilicity. Collectively, these properties favor passive membrane permeation and reduce reliance on carrier-mediated transport for intracellular drug accumulation. In in vitro CML models, ponatinib appears relatively unaffected by ABCB1- and ABCG2-mediated transport and can additionally inhibit the transport activity of both efflux pumps [135,139]. Thus, ponatinib may not only retain antileukemic activity in cells with increased expression of efflux transporters but may also partially counteract ABC-mediated multidrug resistance associated with earlier-generation TKIs.

#### 4.2.2. Activation of Downstream or Alternative Signaling Pathways

A major mechanism of BCR::ABL1-independent resistance involves adaptive rewiring of cellular signaling networks that ultimately uncouples leukemic cell survival from BCR::ABL1 kinase activity. Under prolonged therapeutic pressure, oncogenic signaling may be restored downstream of BCR::ABL1 or rerouted through alternative pathways, allowing leukemic cells to maintain survival despite effective inhibition of the primary oncogenic driver [140,141]. Depending on the pathways involved, this signaling plasticity may variably diminish the therapeutic response to TKI monotherapy and create distinct therapeutic vulnerabilities that can be exploited through rational combination strategies.

- SRC family tyrosine kinases (SFKs)

Among the earliest adaptive changes emerging during prolonged TKI treatment is the activation of SRC family kinases (SFKs), including Lyn, Hck, and Fyn. Of particular clinical relevance is Lyn, whose increased expression and activity have been reported in patients with imatinib-resistant CML in the absence of BCR::ABL1 mutations. Activated Lyn can phosphorylate multiple substrates shared with BCR::ABL1 and maintain signaling through the oncogenic PI3K/AKT, RAS/MAPK, and STAT5 pathways despite effective BCR::ABL1 inhibition [142]. Modulation of SRC activity may partly account for the efficacy of dasatinib in certain forms of BCR::ABL1-independent resistance, as dasatinib targets several SRC family members, including LYN and HCK, in addition to BCR::ABL1. However, this broader kinase-inhibitory profile may also contribute to treatment-related toxicity, as SRC family kinases participate in cardiovascular homeostasis, including endothelial function, vascular integrity, and cardiomyocyte signaling [140,141,142,143]. Thus, SRC inhibition may represent a double-edged sword for current and future TKIs, as it may increase the risk of on-target/off-tumor toxicity.

- RAS/RAF/MEK/ERK signaling pathway

The RAS/RAF/MEK/ERK (MAPK) pathway is a major downstream effector of BCR::ABL1 that regulates leukemic cell proliferation, differentiation, and survival. MAPK activity is, however, embedded within a broader network of crosstalk interactions and, under sustained therapeutic pressure, may become constitutively active independently of BCR::ABL1. Persistent MAPK signaling promotes the expression of cyclin D1, MYC, and other mitogenic factors and engages in extensive crosstalk with the PI3K/AKT, JAK/STAT, and WNT/β-catenin pathways, establishing a wide signaling circuitry that sustains the fitness of leukemic cells during therapy [144]. Reported molecular drivers of MAPK persistence in CML are acquired mutations in KRAS, NRAS and PTPN11, among other pathway regulators. Although relatively uncommon in chronic-phase CML, such alterations have been implicated in BCR::ABL1-independent resistance and appear to be of increasing relevance with disease progression. Indeed, gain-of-function KRAS mutations have been documented in patients with wild-type (WT) BCR::ABL1 and acquired resistance to imatinib therapy [145]. More recently, genomic and transcriptomic profiling of patients with myeloid blast-phase CML linked KRAS and PTPN11 mutations to a lower probability of major hematologic response and inferior progression-free and overall survival. KRAS pathway activation was also reported in patients who lost response to third-generation TKI-based therapy, adding further evidence for the involvement of this pathway in TKI resistance and advanced disease [146].

- JAK/STAT signaling pathway

The JAK/STAT pathway is a central transducer of hematopoietic growth factor and cytokine signaling that is also recruited downstream of BCR::ABL1. In CML, these physiological inputs are amplified through increased secretion of GM-CSF, IL-6, and other pro-inflammatory cytokines by mesenchymal stromal cells (MSCs) within the bone marrow niche, rendering JAK/STAT activity less dependent on BCR::ABL1. These niche-derived signals sustain STAT3 and STAT5 activation, which assume partly overlapping but distinct roles in leukemic cell survival and treatment response. STAT3 predominantly mediates cell-extrinsic, cytokine-responsive survival and proliferative programs and is less tightly coupled to BCR::ABL1 activity, allowing its activation to persist irrespective of BCR::ABL1 kinase inhibition. STAT5, on the other hand, has been more intricately linked to BCR::ABL1 signaling, serving as a direct substrate of the fusion kinase and a key transducer of its leukemogenic activity [147]. Nevertheless, both transcription factors converge on overlapping pro-survival and anti-apoptotic programs, including the induction of BCL2, BCL2L1/BCL-XL, MCL1, BIRC5/survivin, and BIRC3/c-IAP2, raising the apoptotic threshold of leukemic cells. In addition to these effects, STAT3 has also been implicated in the metabolic adaptation of CML cells to TKI therapy by reducing mitochondrial activity and shifting their metabolic dependence toward aerobic glycolysis [148,149]. From a resistance perspective, however, their roles are distinguished less by their downstream effects than by the mechanisms sustaining their activation. In this sense, STAT3 induction predominantly represents a BCR::ABL1-independent survival route, whereas STAT5 activity can be maintained through both BCR::ABL1-dependent and -independent mechanisms. Therefore, concurrent STAT3/STAT5 inhibition may enable more effective suppression of leukemogenic signaling and provide a feasible strategy for overcoming TKI resistance in CML.

- The PI3K/AKT/mTOR signaling pathway

A further pathway of potential relevance to signaling plasticity is the PI3K/AKT/mTOR axis, which is a central regulator of cell survival, growth, metabolism, adaptation to nutrient deprivation and protein synthesis. PI3K/AKT signaling is principally activated downstream of BCR::ABL1; however, aberrant activation can also be maintained through alternative receptor and non-receptor tyrosine kinases, including AXL, SRC, and JAK kinases, or through decreased levels of the negative regulators of Akt—the central node of this pathway [150]. In addition, PI3K mutations may accompany BCR-ABL mutations, resulting in constitutive activation of this pathway and drug resistance against kinase inhibitors. In parallel, Li et al. reported increased phosphorylation of mTOR (70.6%), and its downstream effectors 4E-BP1 (76.5%), and p70S6K (73.5%) in bone marrow cells from patients with CML, providing clinical evidence for constitutive activation of this signaling axis and the potential therapeutic utility of mTOR inhibitors [151].

- The AXL receptor tyrosine kinase

AXL is a receptor tyrosine kinase of the TAM family, whose overexpression has been demonstrated both in imatinib-resistant leukemic cell lines and in patients with acquired resistance to TKI targeted therapy. Functionally, increased AXL activity provides an alternative route for sustaining prosurvival signaling, engaging in a feedback activation of important oncogenic pathways, including PI3K/AKT, MAPK/ERK and NF-κB, that collectively attenuate TKI-induced apoptosis [152,153]. 

AXL overexpression in TKI-resistant CML cells is closely linked to persistent MEK/ERK and PKC signaling. Constitutive ERK1/2 activity promotes PKCα/PKCβ activation, which in turn enhances AP-1-dependent AXL transcription. Accordingly, pharmacological or genetic inhibition of MEK/ERK or PKC reduces AXL expression and restores imatinib sensitivity, whereas PKCα/PKCβ overexpression exerts a desensitizing effect on CML cells. AXL may therefore participate in a positive feedback loop in which persistent MAPK signaling reinforces AXL expression, while activated AXL further engages downstream prosurvival pathways [153,154]. 

The relevance of AXL signaling also extends to the LSC compartment. AXL is preferentially expressed in primary CML CD34+ cells, and its genetic or pharmacological inhibition impairs LSC self-renewal capacity and prolongs survival in preclinical models. This dependency is reinforced by the bone marrow niche, as CML CD34+ cells induce stromal secretion of GAS6, establishing a paracrine GAS6/AXL loop that supports LSC maintenance. GAS6-mediated AXL activation engages both AKT and ERK1/2, with the PI3K/AKT axis promoting β-catenin stabilization through inhibition of GSK3β. Notably, GAS6 retains the capacity to activate AKT following BCR::ABL1 knockdown, indicating that the GAS6/AXL axis can sustain PI3K/AKT signaling independently of BCR::ABL1 [153,155]. AXL can therefore be considered a signaling nexus at the intersection of cell-intrinsic and niche-mediated resistance and a potential therapeutic vulnerability in persistent CML stem cells.

#### 4.2.3. Remodeling of the Bone Marrow Microenvironment

The bone marrow microenvironment (BMME) is increasingly recognized as a key contributor to CML progression, actively shaping stem-cell behavior and creating a permissive niche for LSC persistence and evasion of drug-induced elimination. Through coordinated cellular, metabolic, and immune remodeling, this niche creates a spatially organized cellular and paracrine milieu that promotes leukemic cell quiescence, supports survival under therapeutic pressure, and provides a leukemic reservoir for disease relapse [156].

- Mesenchymal stromal cells and the CXCL12/CXCR4 chemokine axis

MSCs are central to the remodeling of the leukemic BMME. In response to signals derived from malignant leukemic cells, MSCs undergo functional and transcriptional reprogramming accompanied by epigenetic alterations and the acquisition of a pro-inflammatory secretory phenotype characterized by increased production of IL-6, CXCL12, BMP2, BMP4, among other cytokines and growth factors [157,158]. These changes are also accompanied by bone marrow fibrosis, angiogenesis, and extracellular matrix remodeling [159,160], which further privilege leukemic cell growth over normal hematopoiesis. 

A major component of this dynamic interaction with the protective bone marrow niche is the CXCL12/CXCR4 chemokine axis. CXCL12 is a soluble survival factor, secreted predominantly by MSCs, endothelial cells, and osteoblasts, that mediates the chemotaxis of hematopoietic cells within the bone marrow. The overexpression of its ligand CXCR4 on primitive CD34^+^ leukemic cells promotes their homing and retention within CXCL12-rich niches, where they are exposed to stromal and paracrine signals that favor their transition into a quiescent G0 state. The activation of this axis can also induce PI3K/AKT, MAPK/ERK, and JAK/STAT signaling, further strengthening the prosurvival effects of the BMME. Moreover, CXCL12 can directly impede TKI-induced apoptosis through modulation of caspase-3 activity and BCL-XL expression. This microenvironmental dependency of malignant myeloid cells has already been explored as a therapeutic target: pharmacological disruption of the CXCL12/CXCR4 axis with plerixafor (AMD3100) mobilizes leukemic cells from protective bone marrow niches and can restore their sensitivity to TKI treatment [161,162]. Early attempts at clinical translation in CML, however, have been discouraging, and more recent clinical development of CXCR4-directed therapies has largely shifted toward AML and other hematologic malignancies.

An important endogenous negative regulator of this signaling axis is the membrane-bound dipeptidyl peptidase CD26 (DPPIV), which is also preferentially expressed on the surface of LSCs. By cleaving CXCL12, CD26 disrupts CXCL12/CXCR4-dependent retention and promotes the mobilization of LSCs from the bone marrow into the peripheral circulation. Importantly, CD26^+^ LSCs can persist during TKI therapy, including in patients achieving deep molecular responses, implicating this population in residual and advanced-stage disease. Due to its preferential and easily accessible surface expression on LSCs, CD26 serves as a highly attractive therapeutic target—particularly for antigen-directed cellular immunotherapies, a field currently undergoing rapid preclinical development [157,163].

- Paracrine signaling within the bone marrow niche

Beyond chemokine-mediated interactions, the leukemic bone marrow niche provides a complex repertoire of soluble cytokines and growth factors, including IL-6, GM-CSF, FGF2, PlGF, TGF-β, and VEGF, that support leukemic cell survival and persistence [159,164,165]. These paracrine signals engage multiple compensatory pathways, including PI3K/AKT/mTOR, RAS/MAPK, and NF-κB, as well as developmental programs such as WNT/β-catenin, Hedgehog, and Notch. IL-6 is particularly relevant in this context, given its ability to sustain STAT3 signaling, which, as discussed, provides a high fitness advantage to LSCs. Other stromal factors enable additional routes of protection. FGF2, for example, can promote TKI resistance through FGFR-dependent activation of RAS/MAPK signaling and has been detected at increased levels in the bone marrow of CML patients without kinase domain mutations who were resistant to multiple lines of TKI therapy [166]. Similarly, upregulation of the BMP2/4-BMPR1B axis establishes paracrine and autocrine survival cues that maintain and expand primitive CD34^+^ leukemic populations. Under TKI pressure, BMPR1B^high^ cells may progressively establish an autocrine BMP4 loop associated with increased resistance to treatment. Notably, this microenvironmental dysregulation can persist even during remission: bone-marrow BMP2 and BMP4 concentrations remain approximately 2.7-fold higher than in healthy donors, while residual LSCs are enriched for BMP signaling, quiescence and stemness-associated transcriptional programs. Such persistent niche alterations may preserve a reservoir of residual disease capable of driving relapse following treatment withdrawal [167].

- Cell adhesion-mediated drug resistance

Another major mechanism contributing to the protective role of the BMME is cell adhesion-mediated drug resistance (CAM-DR). Adhesive interactions with stromal and vascular compartments anchor primitive CML cells within protective bone-marrow niches and provide complementary survival signals. N-cadherin-mediated adhesion is one such mechanism, promoting β-catenin-dependent signaling and reducing susceptibility to TKI-induced apoptosis [168]. Consistent with its clinical relevance, patient-derived transcriptomic data show progressive upregulation of CDH2 (i.e., the N-cadherin encoding gene) across disease phases, with expression significantly higher in blast- than in chronic disease phase [169]. The VLA-4/VCAM-1 and CD44/E-selectin adhesion axes exert akin protective and prosurvival effects that also appear to be relevant to disease progression. CD44 expression is significantly higher in blast- than chronic-phase CML cells and promotes their E-selectin-mediated retention and quiescence within the vascular niche. The therapeutic prospects of targeting this mechanism are demonstrated in experimental CML models, in which E-selectin blockade with uproleselan (GMI-1271) impaired LSC adhesion to bone marrow endothelium, promoted cell-cycle entry, and enhanced their elimination by imatinib [170,171,172]. Although not yet clinically evaluated in CML, uproleselan has advanced to phase III trials in AML, representing one of the few niche-directed therapeutic strategies to reach clinical evaluation. It may therefore warrant further investigation in CML, particularly in advanced and blast-phase disease.

- Exosomes and immune remodeling

The BMME can also reinforce leukemic cell protection through exosome-mediated intercellular and immune communications. Exosomes released by leukemic and stromal cells carry miRNAs, proteins, and lipids that alter transcriptional and signaling programs in recipient cells and contribute to bone marrow niche remodeling. Under hypoxic conditions, HIF-1α-driven enrichment of exosomal miR-210, a major hypoxamiR, participates in reshaping the BMME by promoting angiogenesis, stemness-associated programs, and metabolic adaptation in recipient stromal, endothelial, and immune cells. Conversely, exosomal miR-126 can exert opposing effects on leukemic cell-niche interactions by downregulating endothelial CXCL12 and VCAM-1 expression and limiting CML-cell endothelial adhesion and chemotaxis [173,174].

In parallel, the bone marrow niche undergoes substantial immune remodeling. Patients with favorable therapeutic responses exhibit expansion of adaptive-like NK-cell populations with higher NKG2C expression, whereas resistant disease is characterized by a predominance of the inhibitory NKG2A^+^ phenotype. Enhanced activity of the HLA-E/NKG2A immune checkpoint axis acts as a functional “brake” on NK-cell competence, suppressing their cytotoxic activity and contributing to an immunosuppressive microenvironment that favors LSC persistence. In addition, the relative expression of activating and inhibitory NK-cell receptors appears to have prognostic relevance in CML: higher NKG2C (KLRC2) expression predicts an optimal TKI response, whereas NKG2A (KLRC1) expression is associated with TKI resistance and early relapse [175]. In light of these findings, increasing attention has focused on immunomodulatory strategies aimed at augmenting immune-mediated antileukemic activity, including concurrent inhibition of BCR::ABL1 and key immunosuppressive checkpoints. NK-cell-based therapies have also been actively explored across various leukemia types, including CML and AML.

#### 4.2.4. Metabolic Reprogramming

Surviving CML stem cells can adapt under therapeutic pressure by reprogramming their metabolism to meet energy and oxygen requirements and sustain rapid proliferation and survival under stressful conditions. These adaptations involve cell-intrinsic changes in energy metabolism, nutrient utilization, mitochondrial function, and redox homeostasis, as well as metabolic and paracrine support from bone marrow stromal cells (BMSCs). 

The major metabolic pathways are initially suppressed by TKI treatment but undergo progressive reprogramming during prolonged exposure. Rapidly proliferating CML cells exhibit a pronounced reliance on Warburg-type aerobic glycolysis, supported by PI3K/AKT/mTOR and HIF-1α signaling. Within the hypoxic bone marrow niche, HIF-1α stabilization enhances glucose uptake and glycolytic flux through upregulation of GLUT1, HK2, and LDHA and may favor a quiescent, less BCR::ABL1-dependent phenotype with reduced susceptibility to TKI-induced elimination [176,177].

Beyond ATP generation, increased glycolytic flux provides metabolic intermediates for the biosynthesis of nucleotides, amino acids, and lipids required to sustain leukemic biomass and proliferation [178]. Prolonged TKI exposure, however, can further reshape this metabolic phenotype, promoting increased glycolytic dependence and lactate accumulation alongside mitochondrial dysfunction and redox imbalance. These adaptations are accompanied by increased expression of carbonic anhydrase 1 (CA1), potentially contributing to acid-base homeostasis and lipid biosynthesis, and α-synuclein, which has been associated with mitochondrial and oxidative stress responses [179].

In contrast, metabolic adaptation within the primitive CML compartment is more complex, as persistent LSCs can retain a pronounced reliance on mitochondrial oxidative phosphorylation (OXPHOS) and increased mitochondrial biogenesis to support their maintenance and self-renewal. TKI exposure of CD34^+^ CML cells increased the expression of electron transport chain genes and was associated with an approximately 3.3-fold higher oxygen consumption than in normal CD34^+^ cells, suggesting a pronounced OXPHOS dependency in LSCs [180].

Metabolic rewiring in CML also extends to amino acid and lipid metabolism, with increased utilization of non-glucose substrates, including glutamine and fatty acids, as alternative carbon and energy sources to support mitochondrial metabolism and replenish tricarboxylic acid (TCA) cycle intermediates. Importantly, the BMME can further reinforce this metabolic plasticity through direct interactions between stromal and leukemic cells. Recent evidence indicates that BMSCs promote a shift toward oxidative metabolism in CML cells, characterized by increased TCA-cycle activity and utilization of glutamine [181]. This metabolic adaptation is mediated, at least in part, by the direct transfer of stromal membrane vesicles carrying TCA-cycle-related proteins to leukemic cells, which enhances their metabolic plasticity, preserves oxidative capacity, reduces oxidative stress, and protects against imatinib-induced apoptosis. Indeed, pharmacological inhibition of the glutamine-TCA axis with telaglenastat attenuated stroma-mediated protection and increased apoptosis when combined with imatinib [182]. Although the roles of OXPHOS and BMSC in metabolic remodeling have been more extensively characterized in AML, these processes may also represent relevant pharmacological targets in CML, where they also appear to be active in persistent LSCs [178].

A distinct metabolic adaptation in primitive CML cells involves CD36-mediated fatty acid uptake. Serving as a scavenger receptor for fatty acids, CD36 is preferentially expressed within the primitive, quiescent CD34^+^CD38^low^ CML population compared to normal bone marrow cells. CD34^+^CD38^low^ CML cells exhibit reduced sensitivity to imatinib, while fatty acids, particularly under conditions of hypoxia and glucose deprivation, provide substrates for mitochondrial β-oxidation to meet cellular energy demands [183]. In co-culture systems with bone marrow adipocytes, hypoxia enhanced fatty acid release from adipocytes and their subsequent uptake by myeloid cells, demonstrating active metabolic crosstalk between leukemic populations and the adipose niche. As demonstrated, pharmacological blockade of CD36 with the irreversible inhibitor sulfo-*N*-succinimidyl oleate (SSO) partially reversed these metabolic adaptations and reduced the expansion capacity of CML stem/progenitor cells. Alternatively, CD36-targeting monoclonal antibodies induced antibody-dependent cellular cytotoxicity against CD36^+^ CML cells in vitro, while exerting minimal effects on normal CD34^+^ populations [178,184]. 

Redox adaptation represents another important component of metabolic resistance. CML cells, including the primitive LSC compartment, can exploit glutathione-dependent antioxidant defenses, cysteine metabolism, and thioredoxin-1 (Trx1) signaling to maintain redox homeostasis and limit ROS-induced cell death. Trx1 is overexpressed in CML, particularly in imatinib-resistant models, whereas its depletion impairs leukemic cell proliferation and colony-forming capacity. A related vulnerability involves the cystine/glutamate antiporter xCT (SLC7A11), which supplies cystine for glutathione synthesis. Pharmacological inhibition of xCT, including with sulfasalazine, can deplete intracellular glutathione, impair GPX4-dependent detoxification of lipid peroxides, and promote ferroptotic cell death [185,186,187]. Again, while these mechanisms have been more extensively studied in AML, their relevance to redox adaptation in CML is also well established, particularly during progression to blast phase.

#### 4.2.5. Epigenetic Mechanisms of CML Persistence and Progression

Epigenetic dysregulation represents another major aspect of CML persistence and progression, reshaping chromatin organization and transcriptional programs that regulate LSC self-renewal, differentiation, apoptosis, and maintenance of a resistant phenotype [188]. A prominent example is the aberrant regulation of Polycomb repressive complexes (PRC). In CML LSCs, PRC2 is characterized by increased expression of its catalytic subunit EZH2 and associated components SUZ12, JARID2, and PHF19, accompanied by reduced EZH1 expression. The resulting transcriptional silencing includes pro-apoptotic genes such as ARF, PERP, BIM, BOK, and PUMA, helping to maintain an apoptosis-resistant LSC state [188,189]. PRC1-associated BMI1 similarly contributes to leukemic self-renewal and clonal maintenance; its expression is increased in CD34^+^ cells from chronic-phase CML and rises further during progression to accelerated and blast phase, while high levels at diagnosis have been associated with imatinib resistance and inferior survival [190].

Other chromatin and transcriptional regulators have also been reported to contribute to the LSC state. SIRT1, a NAD^+^-dependent histone deacetylase overexpressed in CML CD34^+^ cells, is activated downstream of STAT5 and deacetylates p53 and FOXO1, suppressing their transcriptional and pro-apoptotic functions. SIRT1 also modulates DNA-repair proteins and may promote genomic instability and the acquisition of secondary mutations, including BCR::ABL1 kinase-domain alterations [191]. 

Epigenetic adaptation may also arise during therapy. BCL6, expressed at relatively low levels in TKI-naïve CML cells, is induced following TKI exposure and represses the ARF-p53 axis and cell-cycle regulators, including CDKN1A and CDKN1B. Cooperative repression of pro-apoptotic programs by BCL6 and PRC2 may further support LSC persistence during TKI treatment [192,193].

Aberrant DNA methylation is also implicated in the evolution from chronic to blast phase CML. Promoter hypermethylation can silence tumor-suppressor and pro-apoptotic genes, including DAPK1, CALCA, CDKN2A, CDKN2B, and BIM [188,194]. Reduced BIM expression has been reported in approximately 36% of patients with chronic-phase CML and associated with suboptimal responses to imatinib, with promoter hypermethylation implicated as one underlying mechanism. DNA methylation also intersects with non-coding RNA regulation through the miR-29-DNMT1-SP1-CD70/CD27 axis. Reduced miR-29 expression in persistent LSCs maintains CD70 promoter hypomethylation and increased CD70 expression, activating CD27-dependent survival signaling via the Wnt/β-catenin axis and promoting resistance to TKI therapy [188,195].

Epigenetic dysregulation may additionally accompany clonal evolution. Mutations in ASXL1, a regulator of chromatin remodeling, occur in both chronic- and blast-phase CML and may identify clones with enhanced evolutionary potential. At diagnosis, ASXL1 mutations are detectable in approximately 7% of patients with chronic-phase CML and independently predict a lower probability of achieving major molecular response, shorter survival, and an increased risk of subsequently acquiring BCR::ABL1 kinase-domain mutations resistant to TKI therapy [188,196].

## 5. Clinically Exploited Strategies for Overcoming TKI Resistance

Contemporary therapeutic strategies for overcoming resistance in CML are progressively moving beyond conventional approaches based on dose escalation or switching to newer-generation TKIs toward rationally designed combination therapies that simultaneously target distinct mechanisms and levels of drug resistance.

The present section explores therapeutic avenues with established or emerging clinical relevance, including the development of structurally improved TKI analogues, dual-site targeting of BCR::ABL1, concomitant inhibition of downstream signaling pathways, and restoration of apoptotic responsiveness. These strategies aim not only to restore therapeutic sensitivity, but also to restrict clonal evolution, eliminate persistent leukemic cell populations, and promote deeper and more durable molecular responses.

### 5.1. Next-Generation and Clinically Advanced BCR::ABL1 Inhibitors

Key milestones in the evolution of ATP-competitive BCR::ABL1 inhibitors include the development of nilotinib, dasatinib, and ponatinib, which illustrate the sequential application of structure-activity relationship (SAR)-guided optimization to overcome the limitations of the prototype drug. In parallel, efforts continue to develop novel ATP-competitive TKIs with activity against T315I and potentially improved safety profiles, with several investigational molecules demonstrating promising preclinical and early clinical efficacy. The most clinically advanced among them is olverembatinib (HQP1351), a third-generation BCR::ABL1 inhibitor developed and approved in China through structure-guided drug design aimed at overcoming T315I-mediated resistance. Olverembatinib emerged from a scaffold-hopping strategy designed to optimize hydrogen-bond donor-acceptor interactions and spatial occupancy within the BCR::ABL1 ATP-binding pocket. Compared with ponatinib, olverembatinib establishes additional interactions within the kinase binding site that enhance binding affinity and complex stability. These structural properties enable potent inhibition of both native and T315I-mutant BCR::ABL1 variants. Olverembatinib also targets FLT3, KIT, and several other kinases and has demonstrated substantial clinical efficacy with an acceptable safety profile in patients with T315I-positive CML refractory to standard therapy [197]. 

The clinical development of olverembatinib continues to expand across BCR::ABL1-driven hematologic malignancies and beyond. Ongoing studies include its evaluation as monotherapy in both newly diagnosed and previously treated CML at different stages. The phase III POLARIS-2 trial (NCT06423911) is evaluating olverembatinib in patients with CP-CML previously treated with at least two TKIs, including a separate cohort of patients harboring the T315I mutation [198]. In advanced and blast-phase disease, olverembatinib is also being investigated in combination regimens, including with venetoclax and azacitidine (NCT06757855), decitabine and the BCL-2 inhibitor lisaftoclax (NCT06401603), and with azacitidine and venetoclax (NCT06390306) (Table 2) [199,200,201]. The mechanistic basis for these triple combinations lies in the concurrent targeting of several complementary drivers of leukemic persistence: BCR::ABL1 signaling, BCL-2-mediated survival, and aberrant epigenetic regulation.

Beyond CML, olverembatinib is being actively investigated in Ph+ ALL, most notably in the part A, single-arm phase III POLARIS-1 trial (NCT06051409), where the drug has been evaluated in combination with chemotherapy. Preliminary findings indicate that approximately 65% of patients treated with the olverembatinib-based regimen achieved minimal residual disease (MRD) negativity, providing encouraging early evidence of its clinical efficacy [202]. Several other early-phase studies are ongoing (NCT05931757 and NCT06481228), evaluating olverembatinib in combination with blinatumomab or chemotherapy, including sequential treatment strategies incorporating CAR-T-cell therapy in newly diagnosed adults with Ph^+^ B-cell ALL [203,204]. Its therapeutic potential is also being extended to Ph-like ALL harboring ABL-class fusions (NCT07454226), broadening its application across other ABL-driven leukemias [205]. Importantly, the clinical development of olverembatinib has moved beyond hematologic malignancies, with the phase III POLARIS-3 trial (NCT06640361) evaluating its efficacy and safety in patients with succinate dehydrogenase (SDH)-deficient gastrointestinal stromal tumors [206].

Besides olverembatinib, several next-generation BCR::ABL1 inhibitors have entered clinical development, reflecting continued efforts to improve activity against resistant kinase variants while optimizing selectivity and tolerability. Among the ATP-site inhibitors, ELVN-001 is currently undergoing phase I clinical evaluation in previously treated CML (NCT05304377; NCT06787144) [207,208], whereas TGRX-678 has progressed from phase I studies to a registered phase III trial in heavily pretreated CP-CML (NCT07489755) [209]. In parallel, the clinical validation of myristoyl-pocket inhibition by asciminib has stimulated the development of next-generation allosteric inhibitors, including TERN-701, currently evaluated in the phase I/II CARDINAL trial (NCT06163430) [210], and HS-10382, which has entered phase I clinical evaluation in resistant or intolerant CML (NCT05367700) [211]. The key ongoing and registered clinical trials evaluating olverembatinib and other emerging BCR::ABL1 inhibitors are summarized in Table 2.

**Table 2:** Clinical development of olverembatinib and emerging next-generation BCR::ABL1 inhibitors.

Agent	Mechanism/ Inhibitor Class	Clinical Trial	Phase	Disease Setting/Population	Treatment Strategy	Ref.
**Olverembatinib**	Third-generation ATP-site BCR::ABL1 TKI; Multikinase/BCR::ABL1 TKI with activity relevant to SDH-deficient GIST	**POLARIS-2 (NCT06423911)**	III	CP-CML previously treated with ≥2 TKIs; includes a separate T315I-mutant cohort	Olverembatinib monotherapy	[198]
		**NCT06757855**	I/II	Ph^+^ CML in blast phase	Olverembatinib + venetoclax + azacitidine	[201]
		**NCT06401603**	I	Advanced CML and Ph^+^ AML	Olverembatinib + decitabine + lisaftoclax	[199]
		**NCT06390306**	N/A	CML in myeloid blast phase	Third-generation TKI, including olverembatinib, + azacitidine + venetoclax	[200]
		**POLARIS-1 (NCT06051409)**	III	Newly diagnosed Ph^+^ ALL	Olverembatinib + chemotherapy vs. investigator-selected TKI + chemotherapy	[202]
		**NCT05931757**	II	Newly diagnosed adult Ph^+^ ALL	Olverembatinib + blinatumomab	[203]
		**NCT06481228**	N/A	Newly diagnosed adult Ph^+^ B-ALL	Olverembatinib-based therapy incorporating chemotherapy and sequential CAR-T-cell treatment	[204]
		**NCT07454226**	II	Ph-like ALL harboring ABL-class fusions	Olverembatinib-containing combination regimen	[205]
		**POLARIS-3 (NCT06640361)**	III	SDH-deficient GIST	Olverembatinib monotherapy	[206]
**ELVN-001**	Highly selective ATP-site BCR::ABL1 inhibitor	**NCT05304377**	I	Previously treated CML	ELVN-001 monotherapy	[207]
**ELVN-001**	Highly selective ATP-site BCR::ABL1 inhibitor	**NCT06787144**	I	Previously treated CML; Japanese clinical development	ELVN-001 monotherapy	[208]
**TGRX-678**	Next-generation BCR::ABL1 TKI	**NCT07489755**	III	Heavily pretreated CP-CML	TGRX-678 monotherapy	[209]
**TERN-701**	Allosteric BCR::ABL1 inhibitor targeting the myristoyl pocket	**CARDINAL (NCT06163430)**	I/II	Previously treated CP-CML	TERN-701 monotherapy	[210]
**HS-10382**	Allosteric BCR::ABL1 inhibitor targeting the myristoyl pocket	**NCT05367700**	I	Resistant or intolerant CML	HS-10382 monotherapy	[211]

### 5.2. Dual-Site BCR::ABL1 Inhibition

The concept of dual-site BCR::ABL1 inhibition has progressively moved from preclinical validation into clinical evaluation (Table 3). The clinical development of asciminib provided the first opportunity to exploit this strategy by combining a myristoyl-pocket inhibitor with conventional ATP-competitive TKIs. Early phase I studies established the feasibility of combining asciminib with imatinib, nilotinib, or dasatinib, providing clinical proof of principle for simultaneous inhibition of two spatially distinct sites within BCR::ABL1 [212].

This approach was further evaluated in the randomized phase II ASC4MORE trial (NCT03578367), which investigated whether the addition of asciminib could improve treatment outcomes in patients with CP-CML who had received imatinib for at least one year but had not achieved a deep molecular response. At week 48, MR^4^·^5^ was achieved by 19.0% and 28.6% of patients receiving asciminib at 40 or 60 mg once daily, respectively, in addition to imatinib, compared with 0% of patients continuing imatinib alone and 4.8% of those switched to nilotinib. The benefit of the combination was sustained with longer follow-up, with higher rates of deep molecular response also observed at week 96. Importantly, the addition of asciminib was generally well tolerated and was not associated with new or worsening safety concerns [213].

The potential of dual-site inhibition is now being explored more extensively, including in more refractory molecular settings and patients harboring compound kinase-domain mutations. The recruiting phase II PONTHIAC trial (EUCT 2025-521126-15-00) is evaluating asciminib plus ponatinib in patients with CML resistant to ponatinib and/or asciminib, with MR2 at six months as the primary efficacy endpoint [214]. In parallel, the phase II ASC2ADVANCE study (NCT07538401) is evaluating asciminib-based second-line therapy in CP-CML, including the addition of dasatinib in patients with high-risk molecular profiles [215]. More recently, dual-site inhibition has shown clinical potential in a patient harboring the highly resistant BCR::ABL1 T315I/E355G compound mutation, which had emerged during ponatinib therapy. The mutant showed reduced sensitivity to ponatinib and marked resistance to asciminib when administered individually. Notably, under a single-patient investigational protocol, the combination of ponatinib and asciminib restored a complete hematologic response within two weeks, despite prior failure of both agents as monotherapies [216].

**Table 3:** Clinical evaluation of dual-site BCR::ABL1 inhibition strategies in CML.

Study/Trial	Phase/Design	Patient Population	Dual-Site Treatment Regimen	Main Findings/Objective	Ref.
**NCT02081378**	Phase I	CML-CP or CML-AP previously treated with ATP-competitive TKIs	Asciminib 40 mg BID + nilotinib 300 mg BID; asciminib 40 or 60 mg QD + imatinib 400 mg QD; asciminib 80 mg QD + dasatinib 100 mg QD	Established the clinical feasibility of combining myristoyl-pocket inhibition with ATP-competitive BCR::ABL1 inhibition and defined doses for further clinical development	[212]
**ASC4MORE (NCT03578367)**	Randomized Phase II	CML-CP treated with imatinib for ≥1 year without DMR	Asciminib 40 or 60 mg QD + imatinib 400 mg QD; comparators: imatinib 400 mg QD or nilotinib 300 mg BID	At week 48, MR^4^·^5^ was achieved in 19.0% and 28.6% of the asciminib combination groups versus 0% with continued imatinib and 4.8% with nilotinib; benefit persisted with longer follow-up	[213]
**PONTHIAC (EUCT 2025-521126-15-00)**	Phase II; recruiting	CML resistant to ponatinib and/or asciminib	Asciminib + ponatinib	Prospective evaluation of combined myristoyl- and ATP-site inhibition in resistant CML; primary endpoint: MR2 at 6 months	[214]
**ASC2ADVANCE (NCT07538401)**	Phase II	Second-line CP-CML, including patients with high-risk molecular profiles	High-risk mutation cohort: asciminib 80 mg QD during cycle 1 → asciminib 80 mg QD + dasatinib 100 mg QD from cycle 2; patients without high-risk mutations receive asciminib 80 mg QD alone	Evaluates a dual-site inhibition strategy in patients at increased risk of resistance	[215]
**T315I/E355G compound-mutant case**	Single-patient investigational protocol	CML with highly resistant BCR::ABL1 T315I/E355G compound mutation after failure of ponatinib and asciminib monotherapy	Ponatinib 15 mg QD + asciminib 40 mg BID (subsequently escalated to 80 mg BID)	Complete hematologic response achieved within 2 weeks, providing clinical proof of principle for dual-site inhibition in compound mutation-mediated resistance	[216]

BID, twice daily; QD, once daily; MR2, molecular response corresponding to BCR::ABL1^IS ≤ 1%; MR^4^·^5^, deep molecular response corresponding to BCR::ABL1^IS ≤ 0.0032%.

### 5.3. Combination Strategies Targeting Compensatory Signaling Mechanisms

Although TKIs remain the cornerstone of therapy, evolving treatment strategies increasingly seek to extend the therapeutic scope beyond direct BCR::ABL1 inhibition. A range of combination approaches are being explored to co-target downstream, parallel and adaptive pathways that contribute to leukemic-cell survival, persistence and therapeutic resistance. Several rationally designed combinations directed toward these mechanisms have progressed to clinical evaluation.

- Co-targeting of BCR::ABL1 and JAK/STAT signaling

JAK/STAT modulation has gained particular interest in CML because of the ability of cytokine-mediated JAK2 activation within the bone marrow microenvironment to sustain prosurvival STAT3 and STAT5 signaling despite effective BCR::ABL1 inhibition [5,217,218]. This mechanistic premise motivated the clinical evaluation of ruxolitinib, a JAK1/2 inhibitor, in combination with TKIs as a strategy to deepen molecular responses and potentially facilitate treatment-free remission.

Early clinical evidence was provided by a phase I study (NCT01702064) evaluating ruxolitinib in combination with nilotinib in 11 patients with CP-CML and persistent molecular disease. The combination was generally well tolerated, and 4 of 10 evaluable patients achieved undetectable BCR::ABL1 transcripts after six months [219]. A separate phase I/II study (NCT01751425) subsequently evaluated ruxolitinib added to ongoing TKI therapy in patients with minimal residual disease; however, the study was terminated after no additional benefit from ruxolitinib was observed [220]. 

More conclusive evidence has emerged from the randomized phase II SWOG S1712 trial (NCT03654768), which compared ongoing TKI therapy with or without ruxolitinib in patients with CP-CML and residual disease. The trial reported that the combined regimen increased the proportion of patients who achieve deep molecular responses and meet guideline-based criteria for TKI discontinuation. At 12 months, MR4.0 was achieved in 46% of patients receiving ruxolitinib plus a TKI compared with 26% receiving TKI therapy alone, while MR^4.5^ rates were 14% and 3%, respectively. At two years, 29% of patients in the combination arm met NCCN criteria for TKI discontinuation compared with 11% in the TKI-only arm, with mild-to-moderate anemia representing the most frequently observed toxicity [221]. The potential of JAK1/2 inhibition to facilitate TFR is also being evaluated in a phase II study (NCT03610971) in patients with CP-CML who relapsed following a prior attempt at TKI withdrawal. In this study, ruxolitinib is administered with TKI therapy for 12 cycles before a second attempt at treatment discontinuation; the trial is currently active but no results have yet been reported [222].

### 5.4. Targeting Apoptotic Escape: TKI-BCL-2 Inhibitor Combinations 

Restoration of apoptotic competence represents another compelling strategy that has progressed to clinical evaluation in CML. Persistent leukemic populations, including LSCs, may evade apoptosis through increased dependence on anti-apoptotic BCL-2 family proteins, particularly BCL-2, BCL-XL and MCL-1, providing a therapeutic basis for combining TKIs with agents targeting the intrinsic apoptotic machinery [223]. 

Clinical experience with combined BCR::ABL1 and BCL-2 inhibition in CML and other Ph^+^ hematological malignancies, however, remains heterogeneous. In a small, randomized phase II study, the combination of dasatinib and venetoclax demonstrated a favorable safety profile and high efficacy in patients with chronic-phase CML, although cumulative molecular response rates were comparable with those observed in the dasatinib monotherapy arm. Longer follow-up is required to determine whether the combination increases the rate of sustained deep molecular response and the likelihood of achieving treatment-free remission [224]. The potential of BCL-2 inhibition to target residual disease is also being explored in the phase II VARIANT study (NCT05701215), in which venetoclax is administered following TKI discontinuation to patients with CP-CML in sustained deep molecular response, with maintenance of treatment-free remission as the principal therapeutic objective [225].

More substantial clinical results have been reported in advanced BCR::ABL1-positive myeloid malignancies, including blast- and accelerated-phase CML and Ph^+^ AML. In a phase II study, the triplet regimen of venetoclax, ponatinib, and the hypomethylating agent decitabine achieved complete remission or complete remission with incomplete hematologic recovery (CR/CRi) in 50% of patients, including one complete remission and nine CRi responses. An additional 30% achieved morphologic leukemia-free state, yielding an overall bone-marrow response rate of 80%. These results were also observed in clinically challenging populations, included heavily pretreated individuals and those with high-risk chromosomal abnormalities [226]. As discussed above, BCL-2 inhibition is also being incorporated into olverembatinib-based combination regimens currently under clinical evaluation in advanced CML.

Clinical evidence supporting synergistic effects between BCL-2 inhibition and BCR::ABL1-targeted therapy is also available in Ph+ acute lymphoblastic leukemia (Ph^+^ ALL). An early phase I/II clinical study in heavily pretreated patients with relapsed/refractory Ph^+^ ALL demonstrated a favorable safety profile and tangible clinical outcomes of the triple combination ponatinib-venetoclax-dexamethasone. Complete remission was achieved in 50% of patients, with all responders attaining a complete molecular response, irrespective of prior treatment with ponatinib or blinatumomab; notably, these responses were maintained throughout the follow-up period [227].

### 5.5. Targeting Immune Checkpoints in Combination with BCR::ABL1 Inhibition

As in many other malignancies, immune-checkpoint dysregulation constitutes an important facet of the immunosuppressive milieu in CML and may contribute to the persistence of residual leukemic populations despite effective BCR::ABL1 inhibition. Increased PD-L1 expression has been demonstrated on myeloid cells from patients with CML, together with PD-1 expression on T cells, with these immunoregulatory features being particularly prominent in patients classified as high risk by the Sokal score [228]. Subsequent studies have further linked persistent or refractory disease with an exhausted T-cell phenotype characterized by increased expression of PD-1 and other inhibitory receptors, providing a biological rationale for combining BCR::ABL1 suppression with immune-checkpoint blockade.

The PD-1/PD-L1 axis has consequently been the principal checkpoint pathway taken into clinical investigation in CML. One of the earliest studies evaluated nivolumab together with dasatinib in patients with previously treated chronic- or accelerated-phase CML. In this phase Ib dose-escalation trial (NCT02011945), 31 patients who had received at least two prior TKIs were treated with dasatinib plus nivolumab at escalating doses. The combination proved feasible from a safety perspective, with no dose-limiting toxicities observed; however, its antileukemic activity was modest and responses were generally not durable. Among previously dasatinib-exposed patients with chronic-phase disease, MMR at 36 months was achieved in 2 of 8 patients receiving nivolumab at 1 mg/kg and 5 of 11 receiving 3 mg/kg, indicating that release of PD-1-mediated inhibition alone was insufficient to consistently overcome established TKI resistance [229]. 

A conceptually different application of PD-1 inhibition is being explored not in overtly resistant disease, but in patients with persistent molecular disease despite otherwise effective TKI therapy. The phase II BLAST MRD CML/EA9171 study (NCT03516279) adds pembrolizumab to stable treatment with imatinib, dasatinib, or nilotinib, with conversion to undetectable MRD/MR^4.5^ as its principal objective. Early results from the initial safety cohort indicated that the combination was generally well tolerated: among the first six patients, toxicity was predominantly low grade, and one patient achieved undetectable MRD after combined treatment [230]. The trial remains active but is no longer recruiting, and definitive efficacy data have not yet been reported.

PD-L1-directed strategies have also reached clinical testing. In the adaptive phase I/II ACTIW study (NCT02767063), avelumab was incorporated into ongoing TKI therapy in chronic-phase patients who had achieved complete cytogenetic response but remained short of a deep molecular response. The design used avelumab at 10 mg/kg every two weeks for up to eight infusions, with achievement of MR4.5 as the principal measure of molecular improvement; however, publicly available trial records do not currently provide definitive results for the avelumab arm [231]. More recently, the ZEROLMC phase Ib/II trial examined first-line bosutinib combined with the anti-PD-L1 antibody atezolizumab in newly diagnosed chronic-phase CML. Unlike the earlier combinations, this study revealed a clinically important safety limitation, resulting in termination due to hepatotoxicity [232].

Beyond PD-1/PD-L1, the HLA-E/NKG2A axis represents an additional inhibitory pathway of particular interest because it directly regulates NK-cell and CD8^+^ T-cell function. Pharmacological blockade of NKG2A with monalizumab can release both NK- and T-cell effector activity and has already entered clinical development in solid tumor and hematological other malignancies, including AML [233]. To date, however, no dedicated clinical study of direct HLA-E/NKG2A blockade in CML could be identified.

## 6. Emerging Experimental Strategies in CML

### 6.1. Experimental Strategies for BCR::ABL1 Targeting

Modern approaches to TKI design remain strongly oriented toward scaffold-hopping approaches, in which replacement of the core molecular scaffold, modification of heterocyclic elements, or alteration of the three-dimensional organization of the molecule generates structurally novel analogues with improved pharmacological properties. This strategy has been extensively explored using pyrazolopyrimidine-, quinazoline-, pyridazine-, purine-, and benzamide-based pharmacophores, with the aim of achieving a more favorable fit within the ATP-binding pocket, enhanced selectivity and retained activity against mutant forms of BCR::ABL1 [234,235,236]. 

Another emerging approach in experimental BCR::ABL1-targeted medicinal chemistry involves the development of hybrid molecules derived from established TKIs, particularly imatinib and nilotinib, incorporating bulky, three-dimensional or natural-product-derived structural motifs. Among the most exploited ligand classes are plant secondary metabolites, including alkaloids, flavonoids, terpenoids, coumarins, and anthraquinones, as well as organometallic, steroidal, and other lipophilic fragments that expand the structural and functional diversity of hybrid TKIs [236,237,238]. Many of these naturally occurring moieties may contribute to broadening the biological profile of the parent compound not only by improving molecular binding to the BCR::ABL1 target, but also by modulation of efflux pumps (P-gp), membrane permeability, oxidative stress, proteasomal degradation, and other mechanisms implicated in drug resistance [237]. To date, such derivatives have been investigated predominantly in in silico and BCR::ABL1^+^ in vitro models, where some have demonstrated activity comparable to or greater than that of imatinib. However, evidence regarding their in vivo efficacy, safety and pharmacokinetic behavior remains limited [238,239,240,241].

### 6.2. Experimental Targeting of Survival Pathways

Beyond the clinically investigated combinations discussed above, additional signaling dependencies have emerged as potential therapeutic vulnerabilities in preclinical CML models. Accumulating evidence with experimental drugs targeting distinct nodes within leukemic survival circuitry may serve as a steppingstone toward novel therapeutic strategies for overcoming BCR::ABL1-independent resistance. Their clinical translation, however, remains to be established and will likely require a thorough molecular stratification to identify the signaling dependencies of individual resistant clones. Figure 4 provides a schematic overview of the complex interaction between major proliferative signaling pathways and their potential for therapeutic targeting.

- *Co-targeting of the MAPK signaling pathway*

The contribution of persistent RAS/MAPK signaling to the maintenance of leukemic cells under BCR::ABL1 inhibition has positioned this pathway as a particularly relevant target for combination approaches. Accordingly, concomitant inhibition of BCR::ABL1 and selected components of the MAPK cascade has been explored as a strategy to counteract adaptive signaling and enhance the antileukemic activity of TKIs.

The acquisition of a resistant phenotype in CML models has been linked to increased expression of PKCη, an upstream activator of CRAF, together with persistent RAS signaling. Notably, in the context of sustained RAS activity, the weak RAF-inhibitory properties of imatinib, nilotinib, and dasatinib may paradoxically facilitate BRAF/CRAF activation, allowing downstream MEK/ERK signaling to persist despite BCR::ABL1 inhibition and, at the same time, creating a therapeutically exploitable dependence on this pathway. Consistent with this concept, pharmacological targeting of MEK with trametinib or selumetinib has produced synergistic antileukemic effects in combination with imatinib or nilotinib in resistant CML cells in vitro, with corresponding suppression of tumor growth in in vivo models [242,243]. Although this strategy has not yet advanced to clinical evaluation in CML, combined MEK and tyrosine kinase inhibition has entered clinical investigation in other malignancies, with encouraging activity reported particularly in solid tumors driven by aberrant BRAF or KRAS signaling [244,245,246].

- *Co-targeting of the PI3K/AKT/mTOR signaling axis*

Although a limited number of early-stage clinical studies have been conducted in hematological malignancies, co-targeting of the PI3K/AKT/mTOR axis in CML remains largely experimental to date. Early clinical experience with rapamycin in a small cohort of patients with imatinib-resistant accelerated-phase CML provided preliminary evidence of mTOR-dependent antileukemic activity, with leukocyte responses observed in four of six patients. However, these responses were generally transient or insufficient, and further development was largely superseded by the emergence of more effective second-generation TKIs [247].

Nevertheless, several PI3K, AKT, and mTOR inhibitors have demonstrated activity in preclinical CML models, either alone or in combination with TKIs. These include the PI3K inhibitors idelalisib and pictilisib, the mTOR inhibitors everolimus and sirolimus, and the allosteric AKT inhibitor MK-2206, which have been reported to enhance TKI-induced apoptosis and reduce the viability of resistant leukemic populations [150,248,249]. Dual PI3K/mTOR inhibitors, such as dactolisib, have also been evaluated in models of acquired ponatinib resistance driven by mTOR activation and have demonstrated the capacity to restore sensitivity to BCR::ABL1 inhibition and induce apoptosis in resistant CML cells [250].

Targeting this axis may also exploit additional adaptive dependencies in leukemic cells. ATP-competitive mTOR inhibition can induce protective autophagy, whereas its concomitant blockade with downstream autophagy inhibitors, such as hydroxychloroquine, enhances the elimination of resistant cells and prolongs survival in xenograft models [250].

### 6.3. Targeting Alternative Anti-Apoptotic BCL-2 Family Proteins

As discussed in the previous section, selective BCL-2 inhibition has already progressed to clinical evaluation in combination with BCR::ABL1-targeted therapy. Experimental evidence, however, suggests that acquired apoptotic resistance in CML is not solely BCL-2-driven but may be sustained by other anti-apoptotic members of the BCL-2 family. Studies using primary patient-derived samples and xenograft models have shown that blast-phase CML LSCs exhibit increased expression of multiple pro-survival BCL-2 partners, including BCL-2, MCL-1, BCL-XL, and BFL-1. Adaptive shifts in dependence among these proteins may sustain LSC survival under TKI pressure and reduce sensitivity to selective BCL-2 inhibitors such as venetoclax. Accordingly, of emerging preclinical interest are combination approaches with dual BCL-2/BCL-XL (navitoclax) and pan-BCL-2-family inhibitors (sabutoclax) [251]. 

Of note, compelling experimental results have been reported with sabutoclax, which antagonizes multiple anti-apoptotic proteins, including BCL-2, BCL-XL, MCL-1 and BFL-1. In blast-crisis CML models, sabutoclax sensitized bone-marrow-niche-resident, quiescent LSCs to dasatinib, reduced the G0-cell population, impaired colony-forming and serial-engraftment capacity and improved survival in xenograft models [251,252]. Harb et al. demonstrated that parallel pharmacological inhibition of BCL-XL and mTOR with navitoclax and TPP242, respectively, promoted BAD activation, markedly enhanced apoptosis in CML-BP cell lines and primary CD34^+^ progenitors while sparing their healthy counterparts, and overcame TKI resistance mediated by the permissive environment [253].

Some clinical evidence, although not specific to Ph^+^ ALL, supports the potential of dual BCL-2/BCL-XL inhibition in acute lymphoblastic leukemia, where the combination of venetoclax with low-dose navitoclax and chemotherapy has demonstrated good tolerability and distinct therapeutic efficacy [254,255]. However, in the CML therapeutic domain, clinical experience with broader-spectrum BCL-2-inhibitors is still scarce.

### 6.4. Targeting Metabolic Vulnerabilities

The metabolic adaptations that sustain CML LSCs under therapeutic pressure also create dependencies that can be exploited pharmacologically. Persistent LSCs display a pronounced reliance on mitochondrial oxidative phosphorylation (OXPHOS), setting them metabolically apart from more TKI-responsive leukemic cells and exposing mitochondrial metabolism as a potential point of therapeutic intervention. 

One such approach involves interference with mitochondrial protein synthesis. Tigecycline, an inhibitor of mitochondrial translation, preferentially depleted LSCs when combined with imatinib in in vitro CML models. This effect extended to in vivo settings, where the combination markedly impaired the ability of human leukemic stem/progenitor cells to engraft in the bone marrow and reduced their disease-propagating capacity [180]. Metabolic perturbation with metformin has yielded similarly encouraging findings: the drug reduced the clonogenic potential of CML CD34^+^ cells and, when combined with imatinib, decreased bone-marrow engraftment of human CD45^+^CD34^+^ cells by approximately 90%, accompanied by a significant prolongation of survival in vivo [256].

A more direct strategy has focused on disrupting OXPHOS, thereby constraining the mitochondrial energy supply on which primitive leukemic populations depend. The thiophene-carboxamide OXPHOS inhibitor NK-128 enhanced the antileukemic activity of imatinib and restricted tumor growth in CML xenograft models. Mitochondrial function can also be perturbed indirectly: lomerizine, a calcium-channel blocker, disrupted mitochondrial Ca^2+^ homeostasis and OXPHOS, increased the sensitivity of primitive CD34^+^CD38^−^ CML cells to imatinib, and reduced LSC burden while prolonging survival in xenograft models [257,258].

Beyond mitochondrial bioenergetics itself, adaptation to the hypoxic bone-marrow environment represents another metabolically relevant dependency of CML stem cells. HIF-1α appears particularly important in this context: its genetic deletion abolishes leukemogenic potential in murine CML models, whereas pharmacological HIF-1α inhibition with agents such as echinomycin and PX-478 induces cell death in both TKI-sensitive and imatinib- or dasatinib-resistant leukemic cell lines [259].

### 6.5. Co-Targeting of Epigenetic Regulators

Beyond the clinically explored combinations with hypomethylating agents outlined above, preclinical efforts are extending epigenetic co-targeting to additional regulatory mechanisms implicated in TKI persistence and LSC maintenance. 

Among these approaches, EZH2 inhibition has generated some of the strongest preclinical evidence. Selective EZH2 inhibitors, including GSK343, GSK126, and EPZ-6438 (tazemetostat), enhanced the susceptibility of primary CML stem/progenitor cells to TKI-mediated elimination, with combined EZH2 and BCR::ABL1 inhibition producing substantial depletion of LSCs in vitro and suppressing their leukemogenic potential in long-term in vivo models while comparatively sparing normal hematopoietic stem cells [260]. More recent findings have broadened the therapeutic relevance of this target by showing that EZH2 inhibition also extensively remodels aberrant mRNA splicing in a cohort of CML patient samples, including through restoration of the tumor-suppressive splicing factor CELF2 [261].

Histone acetylation and deacetylation pathways have long been explored as a means of sensitizing persistent leukemic populations to TKI therapy. Broad HDAC inhibitors such as panobinostat can disrupt LSC quiescence, interfere with HSP90-dependent stabilization of oncogenic proteins, and enhance TKI-induced apoptosis in CML models [262]. More selective interference with deacetylase activity has focused on sirtuin enzymes, such as SIRT1, which is overexpressed in CML stem/progenitor cells. Pharmacological SIRT1 inhibition restores p53 acetylation and transcriptional activity and synergizes with imatinib in eliminating LSCs from both chronic-phase and blast-crisis CML [263]. More recently, the HDAC inhibitor martinostat has shown activity against TKI-resistant CML cells, restored their responsiveness to imatinib and significantly reduced tumor growth in resistant CML xenograft models [264]. 

Therapeutic interference with acetylation-dependent transcription has also been extended to BET proteins, epigenetic readers that translate acetylated chromatin marks into transcriptional output. BET inhibition has demonstrated synergistic activity with BCR::ABL1 TKIs in CML models, while PROTAC-mediated degradation of BRD4 with dBET1 or dBET6 takes this strategy beyond conventional bromodomain inhibition, producing pronounced antileukemic effects in primary CML samples, including blast-phase disease and T315I-mutant models [265]. A distinct avenue of epigenetic intervention involves PRMT5-mediated arginine methylation. Genetic depletion or pharmacological inhibition of PRMT5 reduced the survival and self-renewal capacity of CML stem/progenitor cells and impaired leukemic propagation in vivo, providing a basis for its further evaluation in TKI-based combinations [266].

### 6.6. PROTAC Strategies

The emergence of proteolysis-targeting chimeras (PROTACs) represents a conceptually new therapeutic approach in CML. Unlike conventional TKIs, which inhibit the catalytic activity of BCR::ABL1, PROTACs recruit the fusion oncoprotein to an E3 ubiquitin ligase, promoting its ubiquitination and subsequent proteasomal degradation. Degradation can therefore eliminate both the kinase-dependent and kinase-independent functions of BCR::ABL1. This is particularly relevant because, beyond its constitutive tyrosine kinase activity, BCR::ABL1 can act as a signaling scaffold that supports the assembly and activation of adaptor-mediated pathways involving GAB2, SHP2, and SHC. These non-catalytic functions can contribute to survival signaling, adhesion and chemotaxis and may remain incompletely suppressed by kinase inhibition alone [267,268]. 

PROTACs are heterobifunctional molecules that exploit the endogenous ubiquitin-proteasome system to selectively degrade target proteins. They consist of three principal components: a warhead that recognizes the target protein, a ligand that recruits an E3 ubiquitin ligase, and a chemical linker connecting the two functional moieties. As illustrated in Figure 5, simultaneous binding to the target protein and the E3 ligase results in the formation of a target protein-PROTAC-E3 ligase ternary complex. This induced spatial proximity facilitates the transfer of ubiquitin molecules to the pharmacological target, followed by its recognition and degradation by the 26S proteasome. Subsequent recycling of the PROTAC molecule enables it to participate in additional degradation cycles, defining this mechanism as event-driven, rather than the classical occupancy-driven mode of action characteristic of enzyme inhibitors [267,269,270].

The human proteome comprises hundreds of E3 ubiquitin ligases, yet most PROTAC technologies developed to date predominantly exploit cereblon (CRBN) or von Hippel-Lindau (VHL). For BCR::ABL1, constructs recruiting the cellular/X-linked inhibitors of apoptosis proteins (cIAP1/XIAP), RNF114, FEM1B, and E3 ligases of the N-degron pathway have also been investigated. The expression of individual E3 ligases may vary across tissues, cell populations, and different stages of neoplastic transformation, providing an opportunity to achieve greater context-dependent selectivity. Importantly, the linker is not merely a passive structural element. Its length, rigidity or flexibility, polarity and attachment geometry influence ternary-complex formation, stability, and cooperativity, as well as cellular permeability and the pharmacokinetic properties of the PROTAC. Accordingly, high affinity for the target protein alone does not ensure efficient degradation: constructs incorporating the same target-binding warhead can differ substantially in degradation potency and selectivity depending on linker design and the identity of the E3 ligase recruiter [268,271]. 

This dependence was evident from the first proof-of-concept studies of BCR::ABL1-directed PROTACs. Initial constructs employed ATP-site TKIs, including imatinib, dasatinib, and bosutinib, as target-binding warheads, whereas subsequent optimization generated more potent degraders capable of targeting clinically relevant BCR::ABL1 mutant variants, with some constructs additionally affecting resistance-associated Src-family signaling [269,271]. Representative BCR::ABL1-directed PROTACs and related degradation strategies, along with their principal experimental characteristics, are summarized in Table 4. 

More recent strategies have expanded targeted degradation beyond ATP-site recognition by incorporating allosteric BCR::ABL1 ligands, particularly GNF-5 and asciminib. This approach exploits the spatially distinct myristoyl pocket and has generated degraders with activity against native BCR::ABL1 as well as clinically relevant single and compound mutant variants [271,272,273,274]. Notably, several asciminib- and ponatinib-derived PROTACs retain degradation activity against variants that show reduced sensitivity to the corresponding parent compound, illustrating the mechanistic distinction between occupancy-driven kinase inhibition and event-driven protein degradation [271].

Alternative designs have further advanced this modality by recruiting BCR::ABL1 through regions outside the kinase and myristoyl pockets or by exploiting alternative degradation pathways. These approaches include targeting of the BCR::ABL1 oligomerization domain and activation of the N-degron pathway, which obviate dependence on kinase-domain mutational status [268,275,276]. In parallel, degradation of proteins that sustain compensatory signaling, such as SOS1, may provide a complementary strategy for BCR::ABL1-independent resistance [277].

The combined use of PROTACs and TKIs also represents a rational therapeutic strategy, integrating rapid inhibition of kinase activity with subsequent physical elimination of BCR::ABL1, potentially allowing lower doses of conventional inhibitors to be used. The increased sensitivity to imatinib observed following GMB-475-mediated degradation of BCR::ABL1 provides experimental evidence supporting this strategy [272]. 

Despite compelling preclinical findings, the development of BCR::ABL1-directed PROTACs remains confined to experimental models, and to date no inducer of proteasomal BCR::ABL1 degradation has entered clinical trials. Major limitations include their large molecular size, insufficient solubility, variable subcellular distribution and difficult-to-predict pharmacokinetic properties. Nevertheless, the entry of orally bioavailable PROTACs into clinical trials for other malignancies indicates that these physicochemical limitations can be overcome through rational molecular optimization [267,268].

**Table 4:** Experimental PROTAC-based strategies for targeted protein degradation in CML.

PROTAC/ Strategy	Target-Binding Ligand/Site	E3 Ligase	Experimental Models	Principal Findings, Including Activity Against BCR::ABL1 Variants	Ref.
**DAS-6-2-2-6-CRBN**	Dasatinib/ATP-binding site	CRBN	**In vitro:** K562; non-BCR::ABL1-dependent cell models used for comparison	Efficient degradation of BCR::ABL1 and c-ABL; potent antiproliferative activity in K562 cells.	[269]
**SIAIS178**	Dasatinib/ATP-binding site	VHL	**In vitro:** K562 and engineered BCR::ABL1-mutant models. **In vivo:** K562 xenograft	Potent degradation of WT BCR::ABL1 and the resistant variants G250E, V299L, F317L and F317V; mutants bearing T315I were not efficiently degraded. Significant antitumor activity in vivo.	[271]
**SIAIS056**	Dasatinib/ATP-binding site	CRBN	**In vitro:** K562 and engineered BCR::ABL1-mutant models. **In vivo:** K562 xenograft	Subnanomolar degradation of WT BCR::ABL1 and activity against several resistant variants; no effective degradation of T315I. Antileukemic activity demonstrated in vivo.	[271,278]
**DMP11**	Dasatinib/ATP-binding site	CRBN	**In vitro:** K562 and imatinib-resistant CML cells	Dual target degradation of BCR::ABL1 and Src-family proteins and activity in imatinib-resistant cells.	[279]
**P19P**	Ponatinib/ATP-binding site	CRBN	**In vitro:** K562 and engineered WT/mutant BCR::ABL1 models, including Ba/F3	Degradation activity against T315I, as well as E255V BCR::ABL1 variants.	[270,271]
**GMB-475**	GNF-5/myristoyl pocket	VHL	**In vitro**: K562 and BCR::ABL1-transformed Ba/F3; primary CML CD34^+^ cells and normal CD34^+^ controls; mutant Ba/F3 models	Active against WT BCR::ABL1 and G250E and T315I models. Particularly efficient degradation and antiproliferative activity against G250E and in primary CML CD34^+^ cells.	[272]
**GMB-805**	Asciminib/myristoyl pocket	VHL	**In vitro:** K562. **In vivo:** K562 xenograft model	10-times more potent BCR::ABL1 degradation than GMB-475 and significant in vitro antiproliferative/in vivo activity.	[280]
**LPA81**	Asciminib/myristoyl pocket	CRBN	**In vitro:** WT and engineered mutant BCR::ABL1 models	Effective degradation of native and mutant BCR::ABL1, including clinically relevant TKI-resistant variants; activity in primary CD34^+^ CML cells and in vivo.	[273]
**LPA-81S**	Asciminib/myristoyl pocket	CRBN	**In vitro:** BCR::ABL1^+^ cell lines and mutant models; primary CML CD34^+^ cells/LSPCs. **In vivo:** experimental CML model	Induces more potent degradation of WT BCR::ABL1, single mutants T315I and E255V, as well as compound variants Y253H/E255V and H396R/T315I than parent LPA-81	[274]
**P19As**	Asciminib/myristoyl pocket	CRBN	**In vitro:** K562 and engineered WT/mutant BCR::ABL1 models	Degradation/activity against T315I, E255K, H396R and V468F, importantly retaining activity against the asciminib-resistant V468F variant.	[281]
**SIAIS100**	Asciminib/myristoyl pocket	CRBN	**In vitro:** K562 and engineered BCR::ABL1 single/compound mutant models	Broad mutant coverage, including acsiminib-responsive mutants T315I and E255V, and asciminib-resistant mutations K294E, P223S, T315M, F359V and G671R. It also degraded mutants harboring compound mutations G250E/T315I, Y253H/T315I, and Y253H/E255V	[271,282]
**PMIBcr/Abl-R6**	BCR::ABL1 oligomerization domain	MDM2	**In vitro:** Ph^+^ leukemia cell lines and primary leukemia cells. **In vivo:** T315I-mutant, imatinib-resistant xenograft	Because recognition occurs outside the kinase domain, degradation is not dependent on ATP-pocket binding. Demonstrated activity against T315I-mutant/imatinib-resistant BCR::ABL1 in vivo, accompanied by p53 activation.	[268]
**N-degron PROTACs**	Dasatinib/ATP-binding site	N-degron pathway	**In vitro:** K562. **In vivo:** K562 xenograft	Tunable BCR::ABL1 degradation and suppression of leukemic growth in vitro and in vivo.	[275,276]
**SIAIS562055**	SOS1-directed ligand/SOS1	CRBN	**In vitro:** K562 and KU812; primary BCR::ABL1^+^ CML samples. **In vivo:** K562 xenograft	Degrades SOS1 rather than BCR::ABL1; suppresses RAS/RAC-ERK signaling and enhances TKI activity, providing a strategy directed toward BCR::ABL1-independent resistance	[277]

### 6.7. RNA-Based Therapeutic Strategies

RNA-based therapies represent one of the most advantageous experimental approaches for overcoming drug resistance in CML. Unlike enzyme inhibitors and PROTAC technologies, small interfering RNAs (siRNAs) suppress oncogene expression at the post-transcriptional level. Gene silencing of BCR::ABL1 prevents translation of the fusion transcript and the subsequent production of a functional oncoprotein, effectively eliminating BCR::ABL1-driven oncogenic signaling at its source.

#### 6.7.1. siRNA-Mediated Silencing of BCR::ABL1

RNA interference (RNAi) is a biological phenomenon representing a conserved regulatory mechanism for the post-transcriptional suppression of gene expression, by which genes are “silenced” through inhibition of the translation of their mRNA transcripts [283,284]. 

Small interfering RNAs (siRNAs) are 21–24-nucleotide double-stranded RNA molecules that are unwound by helicase activity, with one strand subsequently incorporated into the RNA-induced silencing complex (RISC). Complementary binding of the siRNA guide strand to the target messenger RNA (mRNA) directs RISC-mediated degradation through Argonaute proteins with endonuclease activity, which catalyze sequence-specific cleavage of the coding transcript [285]. 

The broad applicability of siRNA derives from its ability to selectively suppress the translation of virtually any pathologically expressed protein. In the context of CML, an additional advantage is the possibility of targeting the unique BCR::ABL1 fusion junction, ensuring high selectivity toward leukemic cells while minimally affecting physiological BCR and ABL1 transcripts. At present, however, the evidence base remains limited, with most data derived from in vitro models, a small number of xenograft studies and a single clinical case.

The first experimental applications of RNAi in CML date back to the early 2000s and established the feasibility of siRNA as a tool for selective BCR::ABL1 gene silencing. Synthetic, chemically modified siRNAs targeting the b3a2 fusion junction of the BCR::ABL1 transcript reduced expression of the fusion oncoprotein by up to 90% in K562 cells and primary CML samples and selectively induced apoptosis in BCR::ABL1^+^ leukemic cells [286]. Early plasmid-based shRNA systems likewise demonstrated effective suppression of BCR::ABL1 expression, reduced cell viability and induced apoptosis in K562 cells, further establishing RNA interference as a plausible therapeutic strategy for CML [287]. 

Subsequent in vitro evidence confirmed that siRNA-mediated silencing of BCR::ABL1 suppresses cell proliferation, reduces colony-forming capacity, and induces apoptosis in both TKI-sensitive and TKI-resistant CML cells. Moreover, combined treatment with BCR::ABL1-targeting siRNAs and TKIs has demonstrated pronounced synergistic effects, restoring the sensitivity of myeloid cells to kinase inhibition and overcoming some mechanisms of drug resistance [284]. 

Despite these beneficial findings, the translation of siRNA-based therapy into the clinical management of CML has faced substantial barriers, primarily related to the effective and safe delivery of siRNA in vivo. Owing to rapid enzymatic degradation, poor cellular penetration, and endosomal sequestration of free siRNA molecules, the development of suitable delivery systems has become a major focus of research. Among the various platforms investigated, lipid nanoparticles and other non-viral carriers have emerged as particularly effective approaches for improving siRNA stability, cellular uptake, and biological activity. In particular, lipopolymeric systems based on modified polyethylenimine (PEI) enable efficient intracellular delivery of siRNA into primary CML cells, resulting in significant suppression of BCR::ABL1 expression and colony-forming capacity. Low-dose siRNA treatment reduced BCR::ABL1 mRNA levels by up to 45–50%, while combination with imatinib further enhanced the antileukemic effect [288]. 

Clinical translation of siRNA-based strategies in CML remains largely unexplored. The first and, to date, only reported clinical application of BCR::ABL1-targeting siRNA was described in 2007 in a female patient with relapsed Ph^+^ CML and imatinib resistance associated with the Y253F mutation. The patient had recurrent disease resistant to both imatinib and cytarabine, which were continued during siRNA administration. Notably, the addition of intravenously administered siRNA using a liposomal carrier was followed by a marked reduction in the BCR::ABL1 transcript level after the first administration, from 6.6% to 0.053% on the International Scale within nine days. However, this effect was not reproduced following subsequent administrations, suggesting insufficient therapeutic exposure or the emergence of secondary resistance [289,290]. Nevertheless, this case established the first clinical proof of feasibility for siRNA-based therapy in CML and provided an initial basis for its further development in the setting of therapeutic resistance.

More recently, siRNA-based approaches have been extended to other molecular determinants implicated in the development and maintenance of drug resistance. One emerging target is DUXAP10, an oncogenic pseudogene whose overexpression promotes cell proliferation, disrupts cell-cycle regulation, and suppresses apoptosis, in part through downregulation of the tumor suppressor PTEN. Given the central role of PTEN as a negative regulator of PI3K/AKT/mTOR signaling, siRNA-mediated DUXAP10 silencing restores PTEN expression, suppresses cell proliferation, induces cell-cycle arrest and promotes apoptotic cell death [290].

Another explored molecular target is TRIM22 (Tripartite Motif-Containing 22)—an interferon-induced protein engaged in antiviral defense, immune system and autophagy regulation. siRNA-mediated silencing of this protein is likewise associated with reduced activity of the PI3K/AKT/mTOR signaling axis and alters the expression of key cell-cycle regulators, including CDK4, Cyclin D1, P70S6K, and p53, resulting in suppressed proliferation and induction of apoptosis in K562 cells [290].

#### 6.7.2. Hybrid RNA-Based Technologies

One of the most recent concepts in RNA-based therapeutics in CML involves the development of hybrid systems combining BCR::ABL1-specific siRNA with other regulatory RNA molecules targeting different mechanisms of drug resistance. Particularly favorable results have been obtained by combining BCR::ABL1-targeting siRNA with miR-185 mimics—synthetic analogues of endogenous miR-185 that reproduce its regulatory effects on the expression of genes involved in cell survival and drug resistance. Co-encapsulation of the two RNA molecules in lipid nanoparticles resulted in 65–99% suppression of BCR::ABL1 expression, near-complete elimination of phosphorylated CrkL (a surrogate marker of BCR::ABL1 activity) and a substantial reduction in the viability of TKI-resistant cells, including models harboring the T315I mutation. Pronounced synergistic effects with TKIs have also been observed, with a significant reduction in the colony-forming capacity of CD34^+^ stem/progenitor cells derived from patients who failed to respond to TKI therapy [291].

### 6.8. Adoptive Cell Therapy

Cellular immunotherapy based on chimeric antigen receptors (CARs), which combine antigen recognition with intracellular activation and co-stimulatory signaling, represents one of the most rapidly evolving fields in modern oncohematology. CAR design has evolved through successive generations to improve T-cell activation, persistence and antitumor efficacy. Fifth-generation constructs build on these advances by integrating cytokine-receptor signaling elements, such as a truncated IL-2Rβ domain and a STAT3-binding motif, that link antigen recognition to JAK/STAT activation (Figure 6A).

Despite the remarkable success of CAR-T-cell therapy in B-cell malignancies, its translation to myeloid neoplasms has proved substantially more challenging. The principal challenge for CAR-based therapy in CML is the identification of antigens that sufficiently discriminate leukemic cells and LSCs from normal hematopoietic stem and progenitor cells, thereby limiting the risk of on-target/off-tumor toxicity. Several candidate targets, including IL1RAP, CD26, CD123, and CD25, have emerged from studies of CML stem-cell biology, whereas additional antigens investigated primarily in AML may become relevant to selected cases of myeloid CML-BP following confirmation of their expression on the transformed clone [292] (Figure 6B). The current evidence supporting these emerging immunotherapeutic approaches is summarized in Table 5.

Clinical experience with CAR-T therapy in CML is, by far, limited to a single case. A notable proof of principle was reported in a patient with multiply relapsed lymphoid CML-BP, refractory to sequential treatment with several generations of TKIs, including ponatinib and asciminib, as well as blinatumomab and two allogeneic hematopoietic stem cell transplantations. Administration of CD19-directed CAR-T-cell therapy with tisagenlecleucel resulted in a durable molecular response persisting for at least 12 months without serious adverse events [293]. Although this observation cannot be extrapolated to chronic-phase CML, it demonstrates that an appropriately antigen-positive transformed clone can become amenable to lineage-directed CAR-T therapy [293].

CAR-based strategies have gained particular momentum in AML, providing a broader repertoire of candidate surface antigens potentially relevant to myeloid CML-BP, including CD33, CD123, CLL-1, CD44v6, CD70, NKG2D ligands, and FRβ (Figure 6B). Their applicability to CML-BP remains contingent on antigen expression and an acceptable therapeutic window, particularly for targets such as CD123 that are also expressed on normal hematopoietic populations [294,295]. Another major challenge is intratumoral antigen heterogeneity and antigen escape. Single-antigen CARs may impose strong selective pressure and may eradicate antigen-positive populations while permitting antigen-negative or antigen-low subclones to survive and subsequently drive relapse. This problem has stimulated the development of dual- and multi-target CAR systems with broadened antigen coverage, limiting the probability of therapeutic escape through loss or downregulation of a single molecular target [294,295,296,297] (Figure 6C). A CD33/CLL-1 TanCAR developed by Wang et al. displayed potent in vitro and in vivo antileukemic activity and provides a preclinical rationale for exploring analogous dual-targeting strategies in blast-phase CML (Table 5) [294].

An alternative strategy seeks to extend immune recognition to intracellular leukemia-associated proteins through peptide-HLA complexes. In principle, peptides encompassing the unique BCR::ABL1 fusion junction could provide highly selective targets if efficiently processed and presented by an appropriate HLA allele (Figure 6D). Experience with fusion-derived neoantigens and TCR-based approaches in AML provides proof of concept for this strategy [298], while TCR-like CAR recognition of peptide-HLA complexes, including PR1/HLA-A2, demonstrates the feasibility of targeting intracellular antigens at the cell surface [299]. However, targeting of BCR::ABL1 with TCR-like CARs remains a largely conceptual and forward-looking approach compared to conventional surface-antigen CAR modalities.

Beyond CAR-T cells, CAR-NK and CAR-macrophage (CAR-M) platforms offer complementary properties that may be advantageous in CML. CAR-NK technologies have evolved from earlier strategies based on the adoptive transfer of unmodified natural killer (NK) cells. Multiple clinical trials in CML and AML have evaluated autologous, allogeneic, or induced pluripotent stem cell (iPSC)-derived NK cells, administered either alone or in combination with TKIs, monoclonal antibodies and cytokines. Clinical evidence accumulated to date indicates good tolerability and a favorable safety profile; however, limited persistence in the circulation, low specificity toward LSCs and suppression of NK-cell function by the bone marrow microenvironment restrict the therapeutic efficacy of this former approach [300]. 

These limitations have driven the development of CAR-NK technologies as a next generation of adoptive cellular immunotherapy. Unlike CAR-T cells, CAR-NK therapy combines CAR-mediated recognition of tumor-associated antigens with the intrinsic mechanisms of NK-cell cytotoxicity, including “missing-self” recognition of cells with reduced MHC-I expression, CD16-dependent antibody-dependent cellular cytotoxicity (ADCC), and secretion of IFN-γ and TNF-α. This may enable effective elimination of LSCs with low MHC-I expression—a characteristic feature of advanced-stage CML, and broadens the spectrum of potential therapeutic targets, including CD70 and CD25 (Table 5) [300,301,302,303]. 

Genetically engineered macrophages (CAR-M) have also been explored as an alternative platform for CAR-based therapeutic modalities. Unlike CAR-T and CAR-NK cells, CAR macrophages not only mediate selective phagocytosis of target cells but also function as antigen-presenting cells, capable of promoting a subsequent adaptive antitumor immune response. Given the high expression of CD26 (DPP4) on CML LSCs, this protein is considered a suitable tumor-associated antigen for targeted CAR-M therapy (Table 5) [304].

**Table 5:** Candidate targets and emerging CAR-based immunotherapies as a therapeutic strategy in AML and CML blast-phase disease.

Target/Strategy	CAR Modality	Disease Context	Principal Evidence/Translational Consideration	Ref.
**CD19**	CAR-T	Lymphoid CML-BP	Clinical proof of principle: tisagenlecleucel produced a durable molecular response for ≥12 months in multiply relapsed lymphoid blast-phase CML	[293]
**IL1RAP**	Candidate surface target	CML LSCs	Preferential association with leukemic stem/progenitor populations supports potential antigen-directed targeting; CAR application remains investigational	[292]
**CD123, CD33, CD44v6, CD70, NKG2D, FRβ**	CAR-T/candidate myeloid target	AML-derived rationale for myeloid CML-BP	Expression on leukemic blasts and LSC-enriched populations supports targeting, but expression on normal hematopoietic populations may cause on-target/off-tumor hematotoxicity. Potential applicability depends on expression by the CML-BP clone and the therapeutic window	[294,295]
**CD123/CLL-1**	Bispecific tandem CAR-T	AML; potential relevance to myeloid CML-BP	Dual targeting broadens antigen coverage and may reduce antigen-escape potential; produced pronounced cytolytic effects on CLL-1+ CD123+ leukaemia cell lines and primary AML tumor cells	[295]
**CD33/CLL-1**	Bispecific tandem CAR-T	AML; potential relevance to myeloid CML-BP	Potent activity against AML cell lines and primary blasts, with antileukemic efficacy in murine models	[296]
**CD123/FRβ**	Bispecific tandem CAR-T	AML; potential relevance to antigen-positive CML-BP	Enhanced leukemia-cell recognition and lysis, with synergistic T-cell activation and increased IFN-γ and IL-2 production upon simultaneous antigen engagement.	[297]
**PR1/HLA-A2**	TCR-like CAR	Myeloid leukemia proof of concept	Demonstrates feasibility of recognizing intracellular leukemia-associated antigens after peptide-HLA presentation	[299]
**CD25 (IL2RA)**	CAR-NK92	CML blast crisis/advanced disease	Enhanced degranulation, IFN-γ secretion and selective killing of CD25^+^ leukemic cells; antileukemic activity demonstrated in vitro and in vivo	[302,303]
**CD70**	iPSC-derived CD70-CAR-NK	Myeloid malignancies; potential relevance to CML/CML-BP	Demonstrated antileukemic activity, including against K562 cells, together with elimination of CD70-expressing alloreactive T cells.	[300]
**CD26 (DPP4)**	CAR-M	CML LSCs	Selective recognition and phagocytosis of CD26^+^ CML cells, including T315I-positive cells; reduced leukemic infiltration and prolonged survival in experimental models	[304]

### 6.9. Pharmacokinetic and Nanotechnology-Based Approaches

An important strategy for overcoming drug resistance in CML is the optimization of the pharmacokinetic properties and intracellular exposure of existing therapeutic agents. Suboptimal intracellular concentrations of TKIs may result from impaired cellular transport, increased activity of efflux transporters, or unfavorable pharmacokinetic properties. Accordingly, in recent years, various approaches have been developed to optimize the intracellular delivery of targeted drugs and enhance their bioavailability.

#### 6.9.1. Inhibition of Drug Efflux

One of the best-characterized mechanisms of pharmacokinetic resistance is the overexpression of ATP-binding cassette (ABC) transporters, including ABCB1 (P-glycoprotein, MDR1), ABCG2 (BCRP) and, to a lesser extent, ABCC1 (MRP1). These membrane proteins actively extrude a variety of drug substrates, including TKIs, from the cell, and compromise their subcellular distribution and therapeutic efficacy.

Various efflux pump inhibitors have been evaluated in preclinical studies, including verapamil and cyclosporine A, as well as more selective agents such as elacridar (GF120918) and tariquidar (XR9576). Their combined administration with TKIs increases intracellular drug accumulation, restores sensitivity in resistant cell lines, and induces apoptosis in leukemic cells [305,306]. 

Despite encouraging in vitro and in vivo findings, the clinical utility of these strategies remains limited. The major obstacles include insufficient selectivity, systemic inhibition of physiological transport mechanisms, as well as an increased risk of nonspecific drug–drug interactions. Furthermore, ABC transporter expression varies considerably among individual patients and across different cell populations, and their functional contribution to drug resistance is uneven among the members of the TKI family [139].

#### 6.9.2. Nanotechnology-Based Drug Delivery Systems

Nanotechnology-based approaches represent one of the most rapidly evolving strategies for optimizing therapy in CML. A major objective during the early development of nanotechnology-based drug delivery systems was to improve the pharmacokinetic profile of TKIs by enhancing their solubility, stability and intracellular distribution. Subsequently, the concept of nanocarriers evolved from passive drug delivery systems toward smart, multifunctional platforms capable of selectively delivering diverse classes of therapeutic agents, including TKIs, nucleic acids and immunotherapeutic agents.

Among the most extensively investigated systems are liposomes, polymeric nanoparticles, lipid nanoparticles (LNPs), polymeric micelles, and solid lipid nanoparticles. Owing to their small size, these carriers may reduce the impact of drug-efflux mechanisms through endocytic uptake and sustained intracellular release of their cargo. Preclinical studies have shown that nanoparticle and liposomal formulations of imatinib and dasatinib enhance membrane permeability and provide higher intracellular drug concentrations and more prolonged intracellular retention compared with the corresponding free compounds. In some experimental models, these systems also demonstrate enhanced antiproliferative activity and more effective suppression of leukemic cells at lower drug concentrations [307,308,309]. 

One of the most advanced applications of nanotechnology in CML is the development of targeted delivery systems for precision RNA-based therapies directed against BCR::ABL1. Of particular interest is the combination of CRISPR/Cas9 with polymeric nanocarriers, which may overcome major limitations of gene therapy related to inefficient delivery and off-target effects. In preclinical studies, cationic lipid-assisted PEG-PLGA polymeric nanoparticles loaded with a plasmid-based CRISPR/Cas9 system encoding the Cas9 nuclease and a specific guide RNA (gRNA) directed against the BCR::ABL1 fusion junction achieved selective inactivation of the fusion oncogene without affecting the normal BCR and ABL1 alleles. Intravenous administration of this gene-editing system significantly reduced leukemic burden and prolonged survival in experimental animals, positioning CRISPR/Cas9 as a viable approach for targeted treatment of CML [310]. 

In parallel, lipid nanoparticles are being developed for the systemic delivery of siRNA molecules designed to suppress BCR::ABL1 expression at the translational level. The use of ionizable lipid nanoparticles (DLin-MC3-DMA LNPs) synthesized using microfluidic technology enables highly efficient accumulation of siRNA in the bone marrow and effective intracellular delivery both in vitro and in vivo, without significant toxicity. Reduced BCR::ABL1 levels correlate with a substantial decrease in leukemic burden and improvement in clinical parameters in the evaluated animal models [311]. 

Liposomes and polymeric nanoparticles also provide opportunities for selective targeting of leukemic cells through surface functionalization with antibodies or other ligands recognizing specific cellular markers. Such systems can simultaneously deliver therapeutic and immunostimulatory agents, including CpG oligonucleotides or RNA encoding leukemia-associated antigens, which in turn activate antigen-presenting cells and enhance the CD8^+^ T-cell-mediated antitumor response. In addition, polymeric nanoparticles can be engineered to target CD44 on LSCs and provide controlled release of immunomodulatory molecules, including anti-inflammatory cytokines, capable of modifying the bone marrow microenvironment and mitigating its protective effects on LSCs [309,312,313]. 

Additional opportunities are offered by inorganic nanomaterials, including gold, iron oxide, and silver nanoparticles, which can combine therapeutic and theranostic functions. These materials can be employed for photothermal ablation, magnetically guided drug delivery, and magnetic resonance imaging-based tracking of nanoparticle distribution. Another particularly promising concept is nuclear targeting, in which nanoparticles are functionalized with short amino acid sequences acting as nuclear localization signals (NLSs) to transport cytotoxic agents directly into the cell nucleus, bypassing P-glycoprotein-mediated drug resistance and potentially enhancing therapeutic efficacy in refractory leukemias. Although most nanotechnology-based approaches remain at the preclinical stage, they provide versatile platforms to enhance drug delivery, address pharmacological resistance, facilitate more selective targeting of leukemic stem cells and integrate therapeutic payloads with diagnostic or immunomodulatory functions [313]. 

The major objective of integrating the evidence reviewed above was to enable therapeutic strategies to be aligned with the underlying mechanisms of resistance. While several resistance determinants can already inform clinical management, many of the corresponding therapeutic approaches remain investigational. Their integration into mechanism-guided combination strategies may ultimately provide a more effective means of preventing or overcoming resistance and targeting persistent disease. Table 6 provides a consolidated overview of actionable mechanisms and corresponding treatment strategies.

**Table 6:** Major mechanisms of TKI resistance in CML, their clinical implications and emerging therapeutic strategies. Representative references are provided; additional supporting studies are discussed in the corresponding sections of the text.

Resistance Mechanism	Principal Biological Consequence	Clinical Implication/Clinically Evaluated Approaches	Potential Experimental and Emerging Interventions	Ref.
BCR::ABL1 kinase-domain mutations, including T315I and compound mutations	Impaired TKI binding; mutation-specific cross-resistance; compound mutations may reduce sensitivity even to later-generation inhibitors	Mutation-guided TKI selection; ponatinib or asciminib for T315I-positive disease, according to prior therapy and mutational profile; consideration of allo-HSCT for highly resistant compound mutations. Clinically advanced alternatives include olverembatinib, ELVN-001, TERN-701, and HS-10382.	BCR::ABL1-targeting PROTACs designed to degrade native and mutant BCR::ABL1; BCR::ABL1-directed siRNA as mutation-independent strategies for reducing oncogene expression; experimental next-generation BCR::ABL1 inhibitors.	[69,91,96,112,198,269,284]
**Mutations in the ABL1 myristoyl-binding pocket**	Reduced asciminib binding and impaired allosteric inhibition	Dual-site ATP-site/myristoyl-pocket inhibition is undergoing clinical evaluation, including asciminib-based combinations. Next-generation allosteric inhibitors such as TERN-701 and HS-10382 are in early stages of clinical development.	Myristoyl-pocket-based PROTACs, such as GMB-475 and GMB-805, as well as asciminib-derived degraders, have demonstrated significant experimental activity across diverse resistance models	[33,116,117,212,272,280]
**BCR::ABL1 amplification and overexpression**	Increased target abundance may exceed pharmacologically achievable BCR::ABL1 inhibition, particularly with less potent TKIs	Confirmation of true amplification by cytogenetic/copy-number methods; switch to a more potent second- or third-generation TKI. Persistent or progressive disease requires reassessment for coexisting mutations and clonal evolution.	Direct depletion of BCR::ABL1 rather than catalytic inhibition may be feasible (e.g., PROTAC-mediated degradation or BCR::ABL1-targeting siRNA)	[123,124,125,126,269,284]
**Impaired intracellular TKI exposure**: reduced OCT1-mediated uptake and increased ABCB1/ABCG2-mediated efflux	Insufficient intracellular TKI concentrations despite adequate systemic exposure; particularly relevant to imatinib	Switching from imatinib to TKIs less dependent on active transport, including dasatinib, nilotinib, bosutinib or ponatinib.	Pharmacological inhibition of MDR1-dependent drug efflux (verapamil, elacridar, tariquidar); nanocarrier-mediated TKI delivery and nuclear-targeted delivery systems designed to bypass transporter-mediated resistance.	[127,130,135,137,138,139,305,306,307]
**Activation of alternative/downstream signaling**, including SRC, RAS/RAF/MEK/ERK, JAK/STAT, PI3K/AKT/mTOR and GAS6/AXL	BCR::ABL1-independent maintenance of proliferation and prosurvival signaling despite adequate inhibition of the primary oncogenic kinase	Selection of TKIs with broader kinase activity may partly address selected mechanisms, e.g., dasatinib in SRC-dependent resistance. Combination strategies have entered clinical investigation, including TKI + ruxolitinib for JAK1/2-dependent signaling and persistent disease.	Rational combinations with MEK inhibitors (trametinib, selumetinib), PI3K/AKT/mTOR inhibitors, or AXL-directed inhibitors; simultaneous targeting of related pathways	[140,141,147,153,155,219,242]
**Apoptotic escape and adaptive dependence on BCL-2-family proteins**	Persistence of leukemic cells and LSCs through BCL-2, BCL-XL, MCL-1 or BFL-1-mediated suppression of mitochondrial apoptosis	Several TKI-venetoclax combinations have progressed to clinical evaluation.	Broader inhibition of anti-apoptotic dependencies with navitoclax (BCL-2/BCL-XL), sabutoclax (pan-BCL-2-family).	[223,224,225,226,251,252]
**Bone-marrow niche-mediated resistance and LSC persistence**: CXCL12/CXCR4, cytokines, N-cadherin, VLA-4/VCAM-1, CD44/E-selectin, CD26	Quiescence, protection from TKI-induced apoptosis, retention of LSCs within supportive niches and persistence of residual disease	Some niche-directed strategies have reached limited clinical evaluation, including G-CSF-mediated mobilization/sensitization.	Disruption of niche-mediated signaling and adhesion pathways (plerixafor targeting the CXCL12/CXCR4 axis; uproleselan targeting E-selectin); CD26-targeted adoptive cellular immunotherapies are being explored primarily in advanced- and blast-phase disease.	[156,162,170,171,172,304]
**Metabolic adaptation and hypoxia-driven persistence**	Reduced dependence on BCR::ABL1, increased mitochondrial/OXPHOS dependence, altered redox homeostasis, and survival of quiescent LSCs	A rationale for combining BCR::ABL1 inhibition with agents targeting metabolic dependencies, particularly in persistent or advanced disease; clinical evidence for such strategies in CML is currently lacking.	Experimental targeting of metabolic dependencies: OXPHOS (tigecycline, metformin); mitochondrial Ca^2+^ homeostasis (NK-128, lomerizine); HIF-1α signaling (echinomycin, PX-478); and redox homeostasis.	[178,180,185,256,257,258,259]
**Epigenetic and transcriptional reprogramming**: EZH2/PRC2, BMI1, SIRT1, BCL6	Stable stem-cell and anti-apoptotic transcriptional states; increased LSC persistence, genomic plasticity and reduced apoptotic responsiveness	Epigenetic mechanisms may be particularly relevant in persistent disease and advanced-phase CML, but have limited current impact on therapeutic behavior	Experimental targeting of BMI1, SIRT1, BCL6 and other chromatin/transcriptional regulators; combined TKI and EZH2 inhibition (tazemetostat); RNA-based suppression of resistance-associated regulators.	[188,192,260,263,265,266]
**Advanced clonal evolution and lineage transformation**	Progressive independence from BCR::ABL1 signaling, accumulation of additional chromosomal abnormalities, blast-phase transformation and reduced responsiveness to TKI monotherapy	Requires multimodal therapy approaches; mutation-guided potent TKI therapy may be combined with hypomethylating agents, chemotherapy and/or BCL-2 inhibition. Eligible patients should be considered for allo-HSCT once an appropriate response is achieved.	Adoptive cellular immunotherapy, particularly in blast-phase disease; investigational CAR-T/CAR-NK/CAR-M approaches targeting myeloid/LSC-associated antigens (CD25, CD26, CD123, IL1RAP, CD70).	[69,91,145,226,294,304]

## 7. Future Perspectives

The near-term evolution of precision medicine in CML is likely to be increasingly predicated on the effective integration of actionable molecular findings into therapeutic decision-making. For BCR::ABL1 mutation profiling, broader clinical implementation of the NGS-based approaches discussed in Section 4.1.1 may further strengthen mutation-guided treatment selection, particularly in patients with emerging acquired resistance. Beyond BCR::ABL1, however, the role of broader genomic profiling in routine management of chronic-phase CML remains less well defined. Additional somatic alterations may help delineate clonal evolution and assume greater clinical relevance in blast-phase disease, where they may also inform therapeutic decisions [69,91].

Alongside mutational profiling, quantitative monitoring of BCR::ABL1 transcripts remains central to treatment adaptation. RT-qPCR continues to represent the standard method for routine molecular monitoring, while digital PCR-based approaches such as RT-dPCR offer greater precision at very low transcript levels [69,72]. In an international cohort of 186 patients, a pre-discontinuation BCR::ABL1 level below 0.0023% on the International Scale was associated with a substantially lower probability of molecular recurrence after a first TKI discontinuation attempt. This analytical advantage may prove particularly useful when assessing deep molecular response and complementing established guideline-based criteria for TFR eligibility [314].

Transcriptomic, multi-omic, and machine-learning approaches remain at an earlier translational stage but are increasingly generating clinically testable biomarkers rather than purely descriptive molecular signatures. A recent transcriptome-based machine-learning model identified a 50-gene peripheral-blood signature predictive of sustained two-year TFR, with discriminatory performance retained in an independent validation cohort [315]. The EURO-SKI Biomarker Study similarly illustrates the transition from broad transcriptomic profiling toward focused gene-expression panels that could complement treatment duration, depth of molecular response, transcript type, and other established predictors of successful TFR [316].

Single-cell transcriptomic and multi-omic profiling are other research tools of great translational value. These approaches can resolve molecularly distinct leukemic stem-cell populations, characterize treatment-persistent cellular states, and reveal heterogeneity that is not captured by conventional BCR::ABL1 monitoring [317,318]. Their most realistic near-term contribution may therefore lie in the discovery of biomarkers and therapeutic vulnerabilities that can subsequently be translated into clinically practicable assays [318].

Machine-learning approaches may further facilitate the integration of multidimensional clinical and biological data for individualized prediction of molecular response and TFR [319]. Models incorporating longitudinal BCR::ABL1 response parameters [320], immunological characteristics [321], and transcriptomic signatures [315] have already demonstrated predictive potential, although their clinical utility remains yet to be validated. In the longer term, integration of these computational approaches with molecular and multi-omic data may also contribute to the development of novel targeted and cellular therapies and to predicting the functional consequences of different resistance phenotypes in disease response to therapy [322].

## 8. Concluding Remarks

The transformation of CML into a largely manageable chronic condition stands among the major achievements of targeted therapy, yet acquired resistance and disease progression remain important barriers to durable therapeutic control. As disease evolution introduces additional molecular and cellular dependencies, particularly in advanced and blast-phase CML, effective management increasingly extends beyond BCR::ABL1 inhibition alone. The key challenge will therefore be to identify druggable vulnerabilities and establish their clinical relevance across the course of disease and in the emergence of treatment resistance.

## Figures and Tables

**Figure 1:**
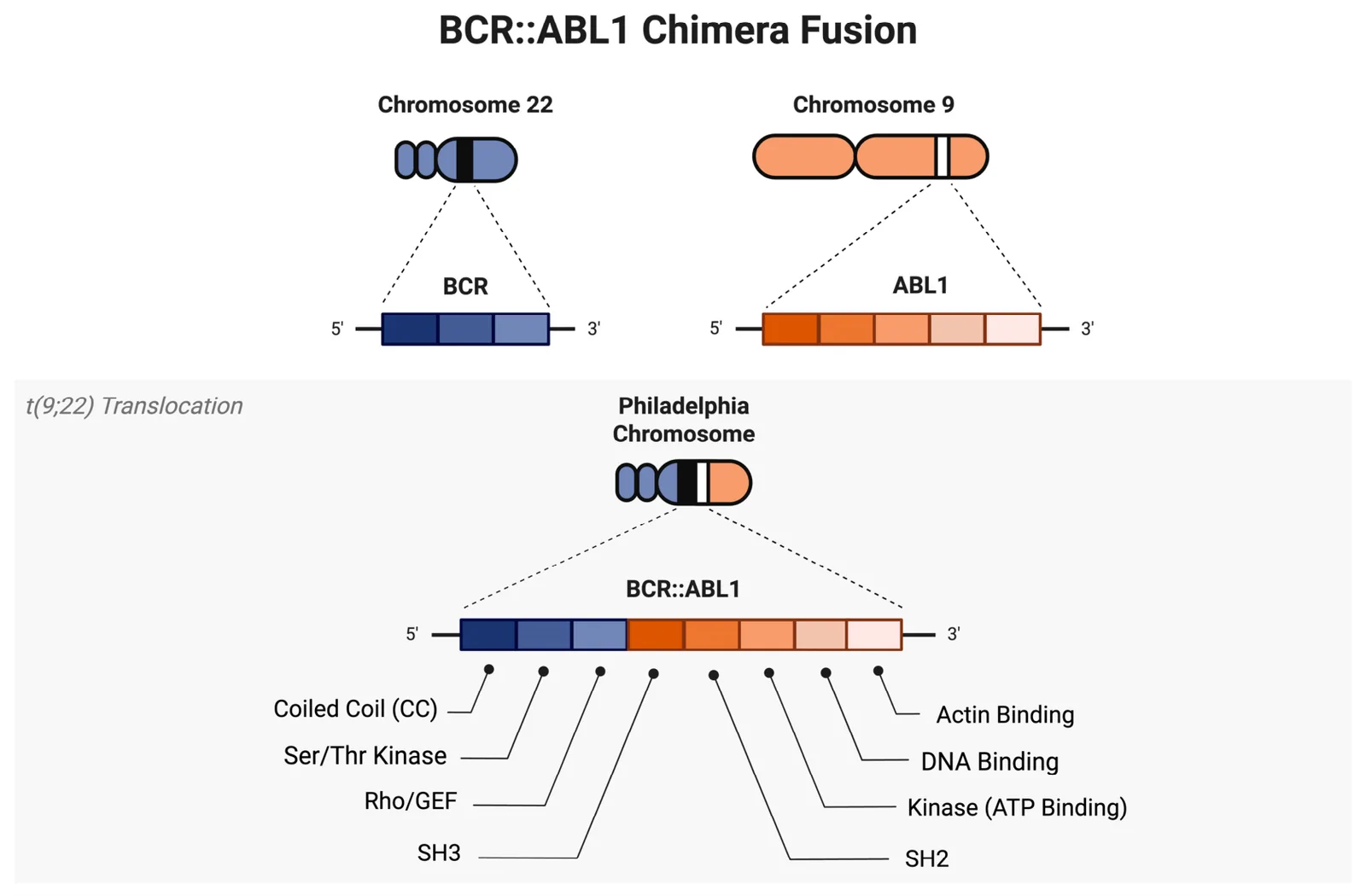
Schematic representation of the t(9;22) translocation and the derivation of the Philadelphia chromosome: a key molecular event in CML pathogenesis leading to the formation of the leukemogenic BCR::ABL1 fusion gene. Created in BioRender. Mihaylova, R. (2026) https://BioRender.com/lmqo6er (accessed on 20 August 2026).

**Figure 2:**
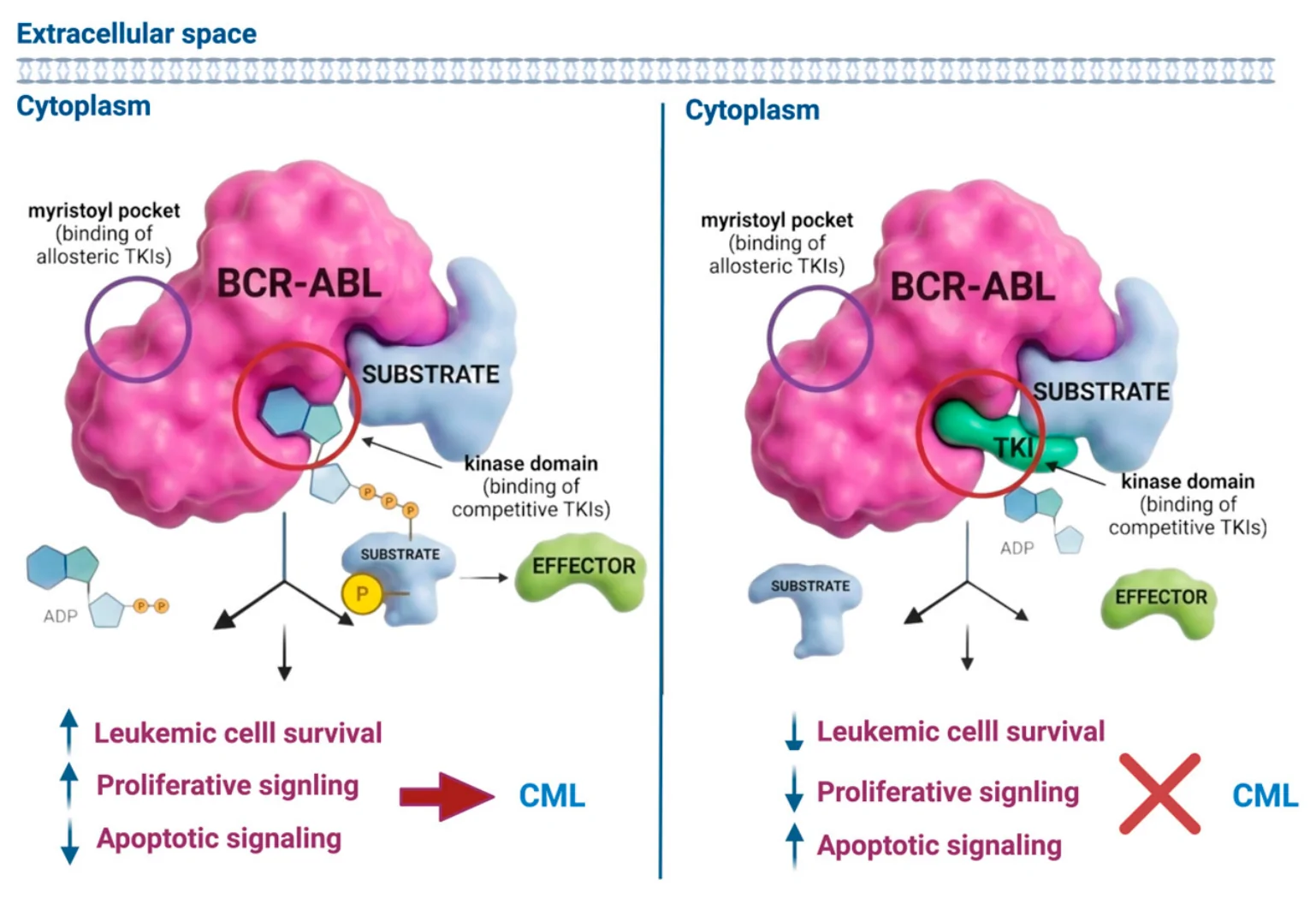
Schematic representation of constitutive BCR::ABL1 kinase activity and its pharmacological inhibition by ATP-competitive and allosteric tyrosine kinase inhibitors. Created in BioRender. Mihaylova, R. (2026) https://BioRender.com/ngztoq3 (accessed on 20 August 2026).

**Figure 3:**
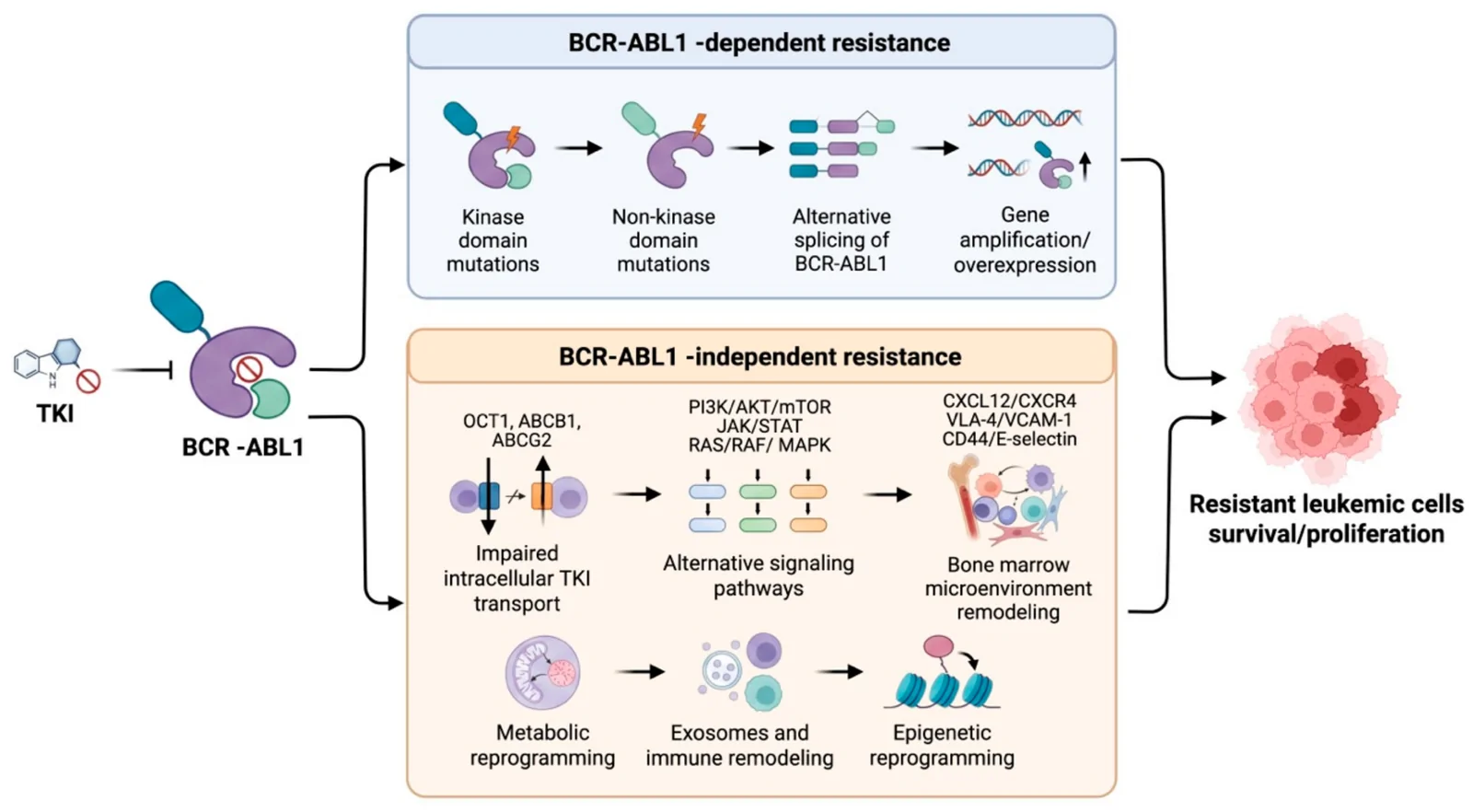
Molecular and cellular mechanisms underlying TKI resistance in CML. BCR::ABL1-dependent alterations may converge with diverse BCR::ABL1-independent adaptive mechanisms to sustain leukemic cell survival and proliferation under therapeutic pressure. Created in BioRender. Mihaylova, R. (2026) https://BioRender.com/zi2drzt (accessed on 20 August 2026).

**Figure 4:**
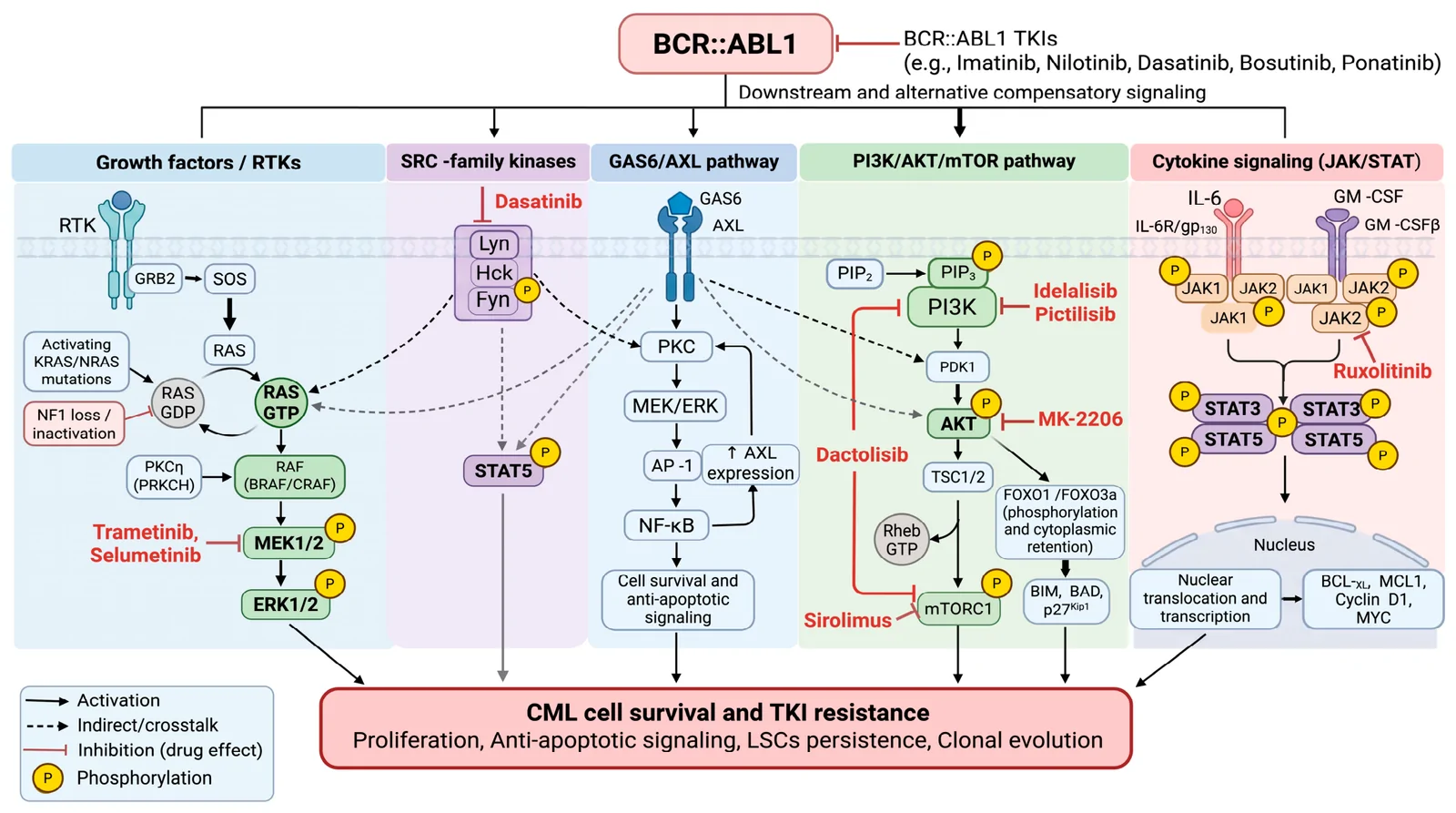
Compensatory signaling in cross-talk pathways contributing to TKI-resistance. Persistent activation of the interrelated RAS/RAF/MEK/ERK, SRC-family kinase, GAS6/AXL, PI3K/AKT/mTOR, and JAK/STAT pathways promotes leukemic cell survival and provides potential targets for pharmacological inhibition. Created in BioRender. Mihaylova, R. (2026) https://BioRender.com/z0qonj0 (accessed on 20 August 2026).

**Figure 5:**
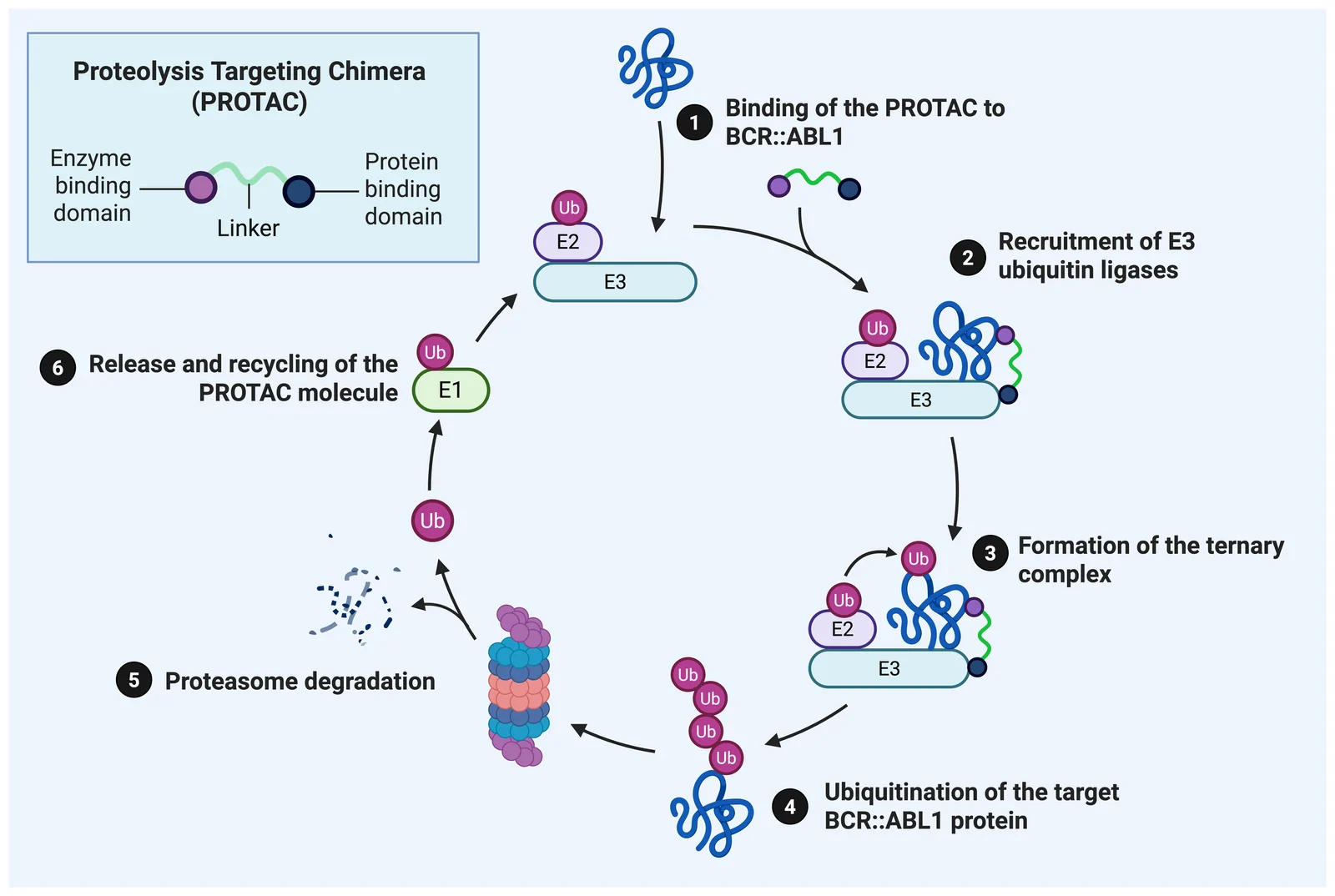
Schematic representation of the mechanism of action of PROTAC-driven targeted degradation of BCR::ABL1. Following recognition of BCR::ABL1, the PROTAC molecule recruits an E3 ubiquitin ligase and induces the formation of a BCR::ABL1-PROTAC-E3 ternary complex. This enables polyubiquitination of the oncoprotein, followed by its recognition and degradation by the 26S proteasome. Upon release, the PROTAC molecule can be recycled and participate in successive cycles of targeted protein degradation. Created in BioRender. Mihaylova, R. (2026) https://BioRender.com/yj6itoa (accessed on 20 August 2026).

**Figure 6:**
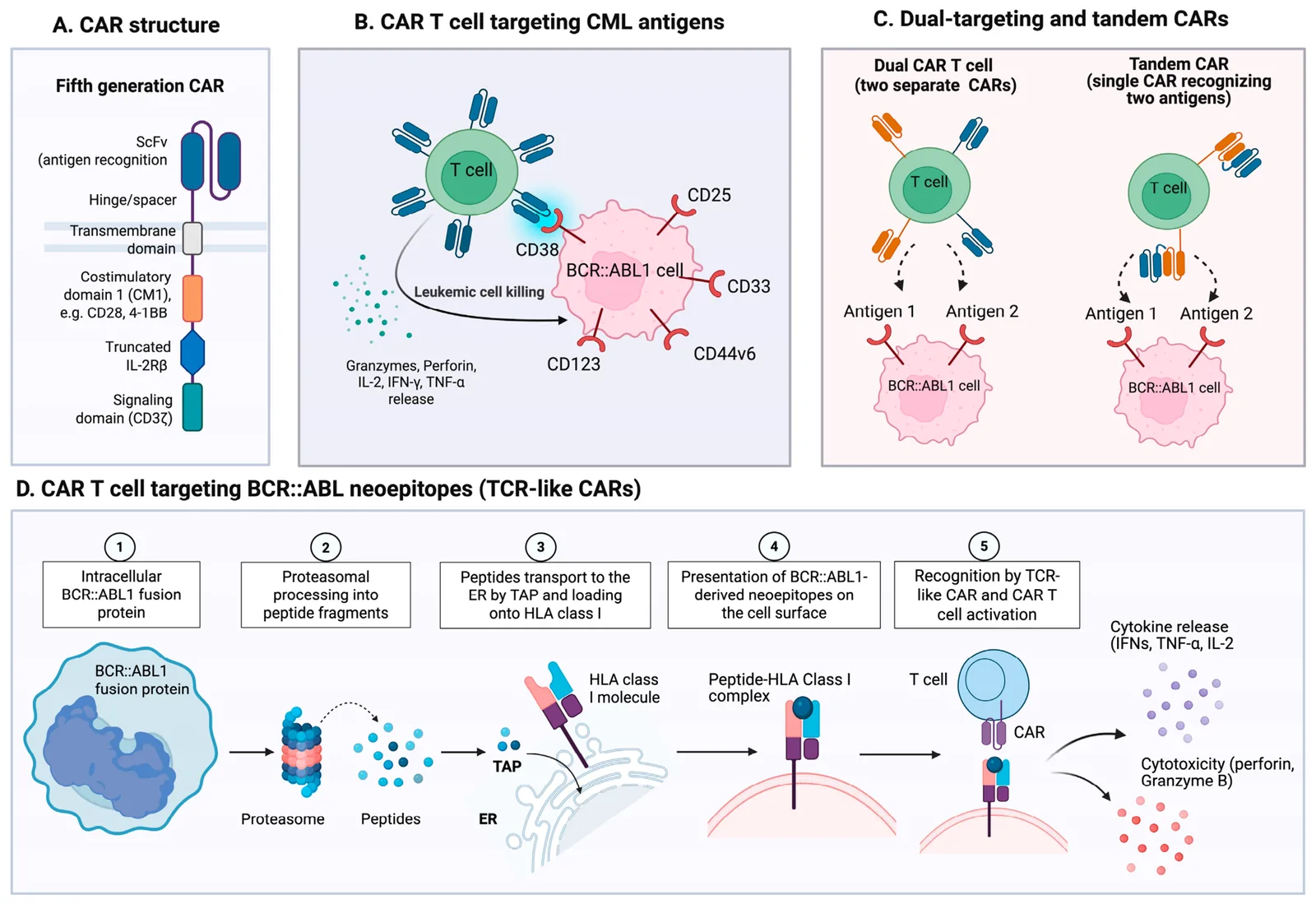
Emerging CAR-based adoptive cell immunotherapies for CML and blast-phase disease. (**A**) Schematic representation of fifth-generation CAR architecture. (**B**) CAR-T-cell targeting AML- and CML-BP-associated surface antigens, including CD25, CD33, CD38, CD44v6, and CD123. (**C**) Dual-targeting strategies based on either two separate CAR constructs or a tandem CAR capable of recognizing two antigens within a single receptor, designed to broaden antigen coverage and reduce antigen escape. (**D**) Conceptual TCR-like CAR targeting of BCR::ABL1-derived neoepitopes. Intracellular BCR::ABL1 undergoes proteasomal processing, followed by transport of peptide fragments into the endoplasmic reticulum, HLA class I loading, and presentation of BCR::ABL1-derived peptide-HLA complexes at the cell surface, where they may become accessible to TCR-like CAR recognition and subsequent T-cell activation. Created in BioRender. Mihaylova, R. (2026) https://BioRender.com/l5grctd (accessed on 20 August 2026).

## Data Availability

No new data were created or analyzed in this study. Data sharing is not applicable to this article.
